# Genome-wide identification of resistance genes and response mechanism analysis of key gene knockout strain to catechol in *Saccharomyces cerevisiae*

**DOI:** 10.3389/fmicb.2024.1364425

**Published:** 2024-02-21

**Authors:** Hong Liao, Qian Li, Yulei Chen, Jiaye Tang, Borui Mou, Fujia Lu, Peng Feng, Wei Li, Jialian Li, Chun Fu, Wencong Long, Ximeng Xiao, Xuebing Han, Wenli Xin, Fengxuan Yang, Menggen Ma, Beidong Liu, Yaojun Yang, Hanyu Wang

**Affiliations:** ^1^Bamboo Diseases and Pests Control and Resources Development Key Laboratory of Sichuan Province, College of Life Science, Leshan Normal University, Leshan, Sichuan, China; ^2^College of Resources, Sichuan Agricultural University, Chengdu, Sichuan, China; ^3^Aba Prefecture Ecological Protection and Development Research Institute, Wenchuan, Sichuan, China; ^4^College of Bioscience and Biotechnology, Hunan Agricultural University, Changsha, Hunan, China; ^5^State Key Laboratory of Subtropical Silviculture, School of Forestry and Biotechnology, Zhejiang A&F University, Hangzhou, Zhejiang, China; ^6^Department of Chemistry and Molecular Biology, University of Gothenburg Medicinaregatan, Gothenburg, Sweden

**Keywords:** catechol, genome-wide identification, response mechanism, *Saccharomyces cerevisiae*, omics analysis

## Abstract

Engineering *Saccharomyces cerevisiae* for biodegradation and transformation of industrial toxic substances such as catechol (CA) has received widespread attention, but the low tolerance of *S. cerevisiae* to CA has limited its development. The exploration and modification of genes or pathways related to CA tolerance in *S. cerevisiae* is an effective way to further improve the utilization efficiency of CA. This study identified 36 genes associated with CA tolerance in *S. cerevisiae* through genome-wide identification and bioinformatics analysis and the *ERG6* knockout strain (*ERG6*Δ) is the most sensitive to CA. Based on the omics analysis of *ERG6*Δ under CA stress, it was found that *ERG6* knockout affects pathways such as intrinsic component of membrane and pentose phosphate pathway. In addition, the study revealed that 29 genes related to the cell wall-membrane system were up-regulated by more than twice, NADPH and NADP^+^ were increased by 2.48 and 4.41 times respectively, and spermidine and spermine were increased by 2.85 and 2.14 times, respectively, in *ERG6*Δ. Overall, the response of cell wall-membrane system, the accumulation of spermidine and NADPH, as well as the increased levels of metabolites in pentose phosphate pathway are important findings in improving the CA resistance. This study provides a theoretical basis for improving the tolerance of strains to CA and reducing the damage caused by CA to the ecological environment and human health.

## 1 Introduction

Catechol (CA) is a major cell growth inhibitor in lignocellulosic hydrolysate, which can inhibit cell growth and limit fermentation productivity of *S. cerevisiae* during alcohol fermentation. CA has received widespread attention due to its high toxicity and carcinogenicity. In 1999, the International Agency for Research on Cancer (IARC) has classified CA as a Group 2B, possible human carcinogen (IARC, 2024). In order to prevent against the hazards of CA to public health and the environment, CA bearing aqueous waste must be treated with efficient, economical, and environmentally friendly techniques before being discharged (Wang et al., 2022). Biodegradation has advantages in terms of low cost, simple operation, and eco-friendliness, and is considered one of the most promising technical methods in removing CA from the waste (Zheng et al., 2019). Previous studies have found that a series of microorganisms possess the ability to degrade CA, such as *Pseudomonas putida*, *Aspergillus awamori*, *Candida parapsilopsis*, *Ralstonia pickettii*, and *Comamonas testosteroni* (Bramhachari et al., 2016). Such discoveries provide the possibility for low-cost and efficient biodegradation of CA, eliminating pollutants from the surroundings.

In the study of microbial degradation mechanisms, it was found that CA was degraded by CA 1,2-dioxygenase (C12O) or CA 2,3-dioxygenase (C23O) through the ortho or meta cleavage, respectively. These enzymes could add two oxygen atoms to the aromatic ring, disrupt the chemical bonds and allow opening of the ring (Whiteley and Lee, 2006). Belonging to ortho lyases, C12O degrade CA into muconate with Fe^3+^ as coenzyme (Tsai et al., 2005). In addition, the C23O, which can catalyze the intermediate cleavage, degrades CA into 2-hydroxymucinous semialdehyde with Fe^3+^ as coenzyme. Among them, muconate is the main raw material in the production of new functional resins, plastics, nylon, agricultural chemicals, and pharmaceuticals (Wang G. et al., 2020). Microorganisms also convert muconate into aliphatic compounds, which enter the tricarboxylic acid cycle, a catabolic processe that support cellular bioenergetics (Whiteley and Lee, 2006; Zeyaullah et al., 2009). However, the large-scale production of mucus acid catalyzed by microorganisms is still unavailable. Yet the development of modern biotechnology has made it possibility to modify the microbial metabolic pathways, in which two strategies stand out. The first is to genetically engineer microorganisms containing CA degradation pathways. The second is to heterologously express the CA 1,2-dioxygenase gene in model organisms (such as *S. cerevisiae*) and optimize it through genetic engineering. In the study of the first strategy, it was found that existing CA-degrading microorganisms are not model organisms. The complex genetic background, unclear metabolic pathways, and unknown genetic transformation systems of non-model organisms are factors that deter scientists from conducting genetic engineering modifications. Therefore, genetic modification of model microorganisms (such as *S. cerevisiae*) is currently the optimum option to achieve high-yield synthesis of muconate. Pyne et al. (2018) obtained a high-yield *S. cerevisiae* strain using CA 1,2-dioxygenase gene transfer and Aro1p engineering degradation technology. However, previous studies have revealed that CA can cause lethal toxicity in people and inhibit the growth and metabolism of microorganisms (Kumar et al., 2005; Rigo et al., 2010; Maini Rekdal et al., 2020). Such harm manifests itself as NADH uncoupling and generation of large amounts of hydrogen peroxide when CA concentration rises as well as CA-induced irreversible damage to biological molecules such as DNA, proteins, and membranes in organisms. In general, during the degradation of CA to muconate in *S. cerevisiae*, CA inhibits the fermentation rate of *S. cerevisiae* and reduces productivity. Therefore, there is enormous potential in mining and engineering CA tolerance-related genes or pathways in *S. cerevisiae* to further improve the fermentation efficiency of *S. cerevisiae*.

Therefore, the optimal CA concentration for inhibiting the growth of *S. cerevisiae* was determined in this experiment, and the gene knockout library in *S. cerevisiae* was cultured in the appropriate concentration of CA, and genes related to CA tolerance were screened using SGAtool software. The enrichment of KEGG and GO were applied for the identification of key pathways related to CA tolerance. In addition, based on the spot test and incubation under CA stress, the function of candidate genes was verified. It was found that the knockout of gene *ERG6* caused extreme sensitivity of *S. cerevisiae* to CA. The response mechanism of *ERG6* gene knockout strain under CA stress was revealed through the observation of subcellular structure and the analysis of transcriptome and metabolome, and the tolerance mechanism mediated by *ERG6* under CA stress was ultimately determined.

## 2 Materials and methods

### 2.1 Strain and culture conditions

The non-essential gene knockout strain library of *S. cerevisiae* used in this experiment was donated by Professor Beidong Liu of the University of Gothenburg. The YPD liquid medium (pH 7.0) was composed of 1% (wt/vol) yeast extract powder, 2% (wt/vol) peptone, and 2% (wt/vol) glucose, and the solid medium was supplemented with 2% (wt/vol) agar. The YPD + geneticin medium was prepared by adding 100 mg/L geneticin to the YPD medium. Agar, peptone, glucose, yeast extract powder, and geneticin (G418) were purchased from Chengdu Wanke Co., Ltd. Before conducting the spot test, the strain (knockout strain) was streaked on a YPD + G418 plate to obtain single colonies, which were inoculated into a 100 mL conical flask containing 30 mL of YPD + G418 liquid medium. The cells were cultured at 30°C and 200 r/min for 18–24 h to obtain cell suspension for the spot test. In the spot test, the cell concentration (*OD_600_*) of the above cultured cell suspension was uniformly calibrated to 0.5, and the cell suspension was diluted stepwise by a 10-fold concentration gradient to obtain different concentrations of cell suspension. The cell suspension was diluted to 5 μL with a row pipette and spotted on a YPD + G418 solid medium containing different concentrations of CA. After 3–4 days of cultivation, photographs were taken. In the liquid culture experiment, the cell concentration (*OD_600_*) of the above cultured cell suspension was uniformly calibrated to 0.25, and the cell suspension was inoculated into a YPD + G418 liquid medium containing different concentrations of CA. At 6 h, 12 h, 18 h, 24 h, 36 h, and 48 h, the cell concentration was determined using a spectrophotometer, and the growth curve was plotted.

### 2.2 Screening and validation of CA sensitive knockout strains

More than 4,400 *S. cerevisiae* gene knockout strains were inoculated on solid plate cultures containing CA through a large-scale cell manipulation platform, and screened for sensitive or tolerant strains using SGAtool software. The CA tolerance or sensitivity screening weight value of ≥0.2 or ≤ −0.2 was used as the standard for tolerance or sensitivity (Cao et al., 2022). If the screening weight value is ≥0.2, it indicates that the knockout gene is a sensitive gene; if the screening weight value is ≤ − 0.2, it indicates that the knockout gene is a tolerant gene. In this study, the genes obtained from the screening were classified using Cytoscape software (Cytoscape 3.9.1) for KEGG and GO enrichment analysis, which facilitated subsequent verification analysis. The activated bacterial solutions of the original strain BY4741 and the gene knockout strains were spotted on YPD + G418 agar plates containing 0 and 1.6 g/L, respectively, using the previously mentioned method, and their growth conditions were observed and photographed for comparative analysis.

### 2.3 Subcellular structural staining

The original strain BY4741 (CK) and the candidate gene knockout strains were cultured overnight in a constant temperature shaker incubator at 30°C and 200 r/min, and the cells labeled as 0 h were collected at this time. Then, in YPD + G418 liquid medium, the original strain BY4741 and the gene knockout strain broth cultures that had been incubated overnight were separately added to YPD + G418 liquid medium containing 1.6 g/L CA (*OD_600_* = 0.8), and cultured for another 3 h at 30°C and 200 r/min, and the cells labeled as 3 h were collected. The collected 0 h and 3 h broths were separately added to 1.5 mL EP tubes (*OD_600_* = 1.0), and the centrifuged broths were stained with the stains (thawed in advance)-Diaminophenylindole (DAPI) (purchased from Shanghai Shenggong Biotechnology Co., Ltd.), Mito Tracker Green FM, ER-Tracker Red dye, Yeast Vacuole Membrane Marker MDY-64 (all purchased from Thermo Scientific), and 2′7′-DCFdiacetate (purchased from Sigma), respectively. Afterwards, changes insubcellular structures were observed in real time using a fluorescence microscope equipped with DIC, GFP, Rhod, and DAPI filters, and the accumulation of ROS, chromatin disorder, mitochondrial damage, endoplasmic reticulum damage, and vacuole damage in the cells were statistically analyzed (Li et al., 2023; Wang et al., 2023).

### 2.4 Sample preparation for transcriptome and metabolome

The cells of BY4741 and ERG6 gene knockout strains cultured overnight were diluted to an *OD_600_* value of 0.3. 20 mL of cells were collected for transcriptome and metabolome analysis, and the two groups were named YOK202W_Q_CA and YML008C_Q_CA. Subsequently, 1.6 g/L CA was added to the culture medium, and 20 mL of cells were collected after 3 h of culture for transcriptome and metabolome analysis, and the two groups were named YOK202W_H_CA and YML008C_H_CA. Three biological replicates were set for each group in the transcriptome analysis. Six biological replicates were set for each group in the metabolome analysis. At the same time the remaining bacterial solution was left continuously cultured during which cell concentration was measured every 6 h, and the growth curve was drawn to determine the impact of gene knockout on the strain tolerance to ensure accurate sampling (or avoid sampling bias).

### 2.5 Transcriptome analysis

Total RNA was extracted using TRIzol reagent (Thermo Fisher, 15596018), and total amounts and integrity of RNA were assessed using the RNA Nano 6000 Assay Kit of the Bioanalyzer 2100 system (Agilent Technologies, CA, USA). FPKM, expected number of Fragments Per Kilobase of transcript sequence per Millions base pairs sequenced, is used to estimate gene expression levels. Differential expression analysis was conducted using the DESeq2 R package. |log2(foldchange)| ≥ 1 (padj≤0.05) was set as the threshold for significantly differential expression. KEGG and GO enrichment analysis of differentially expressed genes (DEG) were performed with the ClueGo program in Cytoscape 3.9.1. All parameters used for RNA-seq are shown in Supplementary material Data Sheet 1.

### 2.6 Metabolomics analysis

In untargeted metabolomic anaylysis, UHPLC–MS/MS analyses of samples were performed using a Vanquish UHPLC system (ThermoFisher, Germany) coupled with an Orbitrap Q ExactiveTMHF mass spectrometer (Thermo Fisher, Germany). The raw data files were processed using the Compound Discoverer 3.1 (CD3.1, ThermoFisher) to perform peak alignment, peak picking, and quantitation for each metabolite. Statistical analyses were performed using the statistical software R and Python. These metabolites were annotated using the KEGG database. All parameters used for metabolomic analysis is shown in Supplementary material Data Sheet 1.

### 2.7 Combined analysis of transcriptome and metabolome

Top10 differential genes and Top5 differential metabolites were screened and mapped to the KEGG pathway database to obtain their common pathway information and determine the main biochemical pathways and signal transduction pathways that are jointly involved in differential metabolites and differentially exprerssed genes. iPath (interactive Pathways Explorer)^1^ is a web-based analysis tool for visualization and analysis of pathways; it summarizes various metabolic pathways in biological systems, with nodes representing various biochemical molecules and lines representing biochemical reactions. The enriched pathways shared by differential metabolites and differential genes were mapped to the iPath website. Based on the above enrichment results, the pathway map of the metabolic pathways was displayed.

## 3 Results and analysis

### 3.1 Screening and spot test of key genes

**Figure 1:**
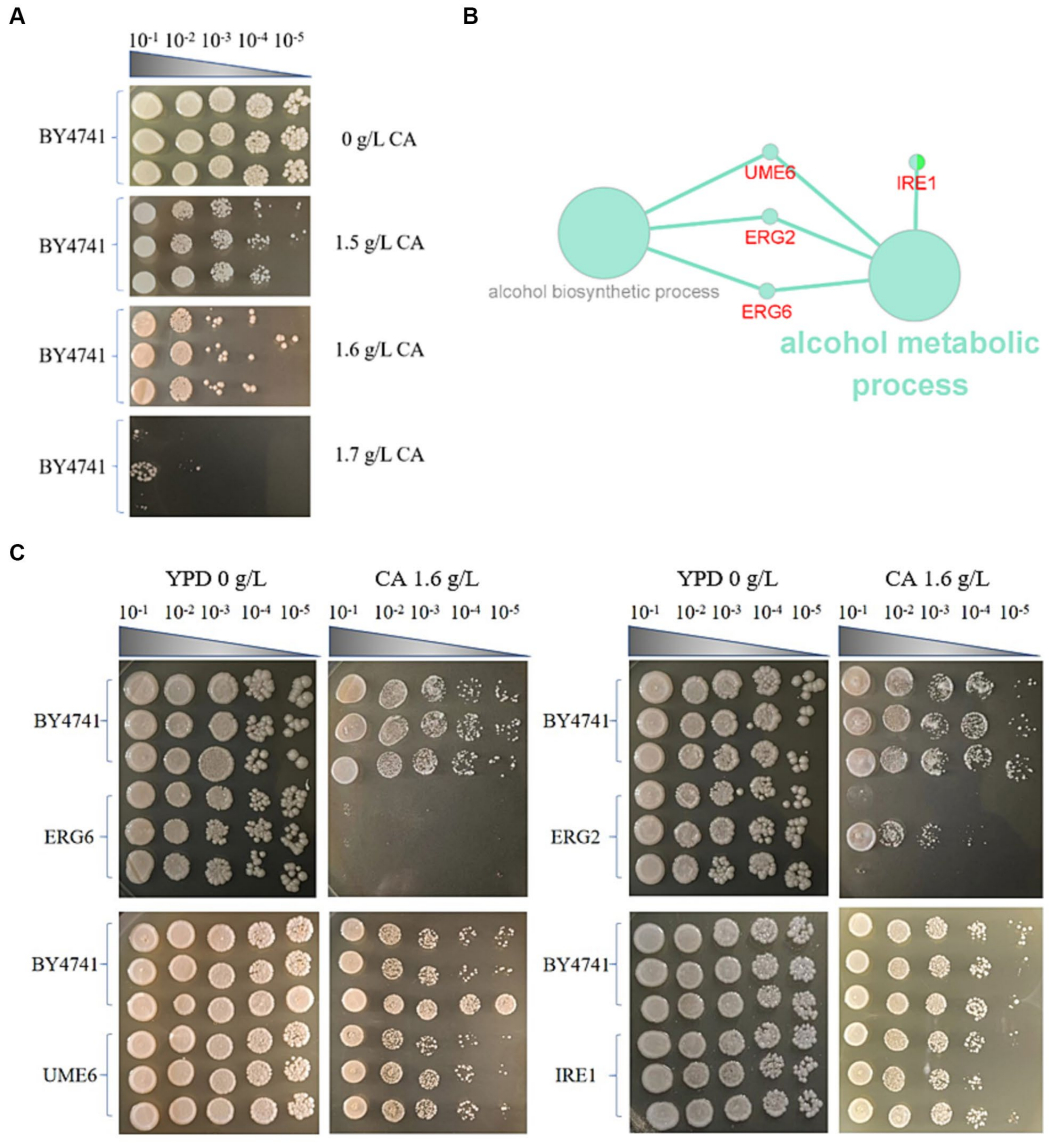
Screening of knockout strains sensitive to catechol (CA) in *S. cerevisiae*. **(A)** Spot test of the strain BY4741 in the YPD medium containing different concentrations of *CA.* **(B)** KEGG and GO enrichment of genes for sensitivity to CA in *S. cerevisiae*. **(C)** Spot test verification of key knockout strains in *S. cerevisiae* in the YPD medium containing 1.6 g/L CA.

Spot test detection was performed in YPD + G418 solid culture medium containing 1.5 g/L, 1.6 g/L, and 1.7 g/L CA (Figure 1A) with no significant inhibition, significant inhibition, and severe inhibition observed, respectively. Therefore, 1.6 g/L is chosen as the most suitable inhibitory concentration. Subsequently 14 non-essential gene knockout colonies (1536-format) were screened on YPD + G418 agar plates containing 1.6 g/L CA. After collecting images of the control and experimental boards, SGAtools was used to analyze the above images and obtain the growth weight values of each knockout strain, as shown in Supplementary Table S1. Screening score set ≥0.2 or ≤ −0.2, a total of 36 gene knockout strains were found to exhibit phenotypic differences with the presence of CA. Among them, 31 gene knockout strains had sensitivity screening weights ≤ −0.2, indicating that these genes are involved in the positive regulation of CA tolerance in brewing yeast. and 6 gene knockout strains had sensitivity screening weights ≥0.2, indicating that these genes are involved in the negative regulation of CA tolerance in brewing yeast.

Using Cytoscape software (Cytoscape 3.9.1), KEGG and GO enrichment analysis were performed on the genes preliminarily screened by SGAtool, and 8 out of 36 genes were found enriched in ethanol metabolism, mitochondrial structural stability pathway, and endoplasmic reticulum composition pathways, respectively. Among them, *ERG2*, *ERG6*, *UME6*, and *IRE1* are enriched in the ethanol metabolism pathway (Figure 1B). *ERG2*, *ERG6*, and *UME6* in the ethanol metabolism pathway are all associated with the processes of alcohol metabolism and alcohol biosynthesis, but *IRE1* only participates in the process of alcohol metabolism. In addition, *ERG2* and *ERG6* are related to the synthesis of sterols. Since a large-scale screening in the early stage of the experiment may inevitably lead to false positives, a spot test was conducted to validate *ERG2*, *ERG6*, *UME6*, and *IRE1* knockout strains (Figure 1C). As shown in Figure 1C, the *ERG6* gene knockout (*ERG6*Δ) strain exhibits the weakest tolerance to CA, with the largest difference compared to the BY4741 standard strain. Therefore, the molecular mechanism of the stress response in the strain *ERG6*Δ under 1.6 g/L CA pressure will be analyzed.

### 3.2 Transcriptome and metabolomic analysis of ERG6 knockout strain under CA stress

**Figure 2:**
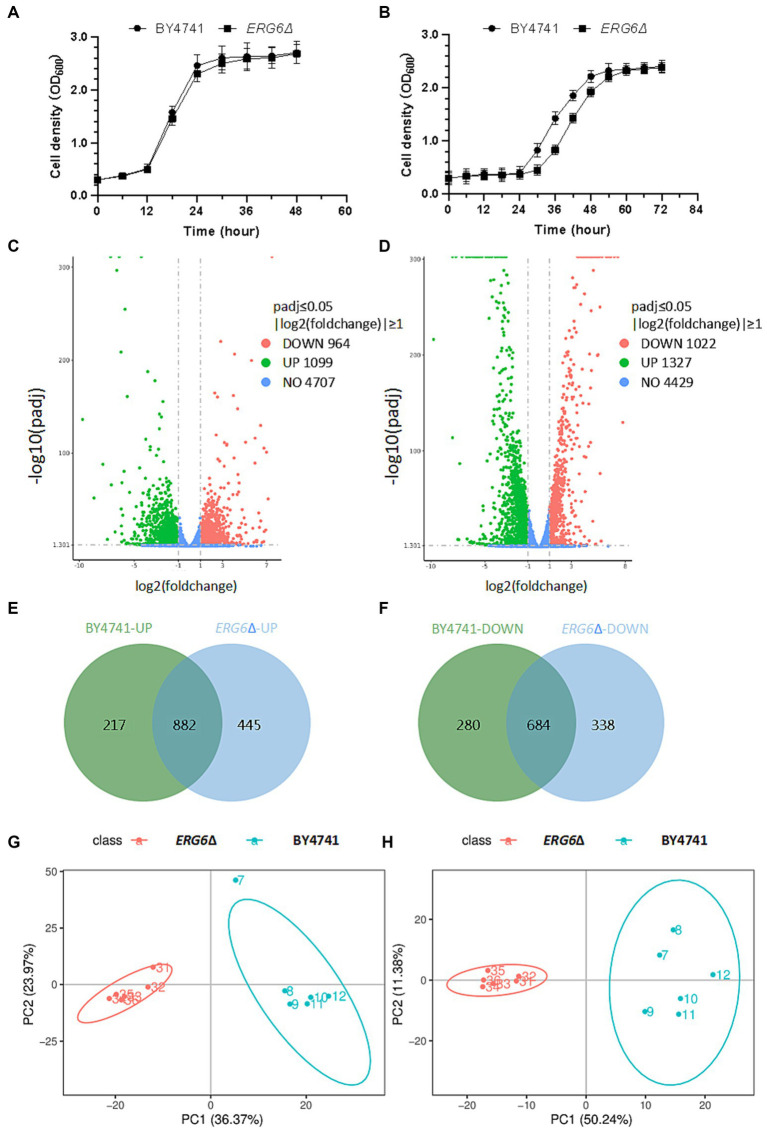
Transcriptome and metabolome analysis of the strain BY4741 and *ERG6*Δ in 1.6 g/L CA condition. Growth curve of the strain BY4741 and *ERG6*Δ in the YPD medium **(A)** and YPD medium containing 1.6 g/L CA **(B)**. Volcano plot of differentially expressed genes in the strain BY4741 **(C)** and *ERG6*Δ **(D)** after CA treatment for 3 h. **(E)** Venn diagram analysis of up-regulated genes in strain *ERG6*Δcompared to the strain BY4741 under CA stress. **(F)** Venn diagram analysis of down-regulated genes in strain *ERG6*Δ compared to the strain BY4741 under CA stress. Principal component analysis of positively charged **(G)** and negatively charged **(H)** metabolites between strain *ERG6*Δ and BY4741.

From the growth curve (Figure 2A), it can be seen that the strain *ERG6*Δ and BY4741 cultured in YPD + G418 medium had similar growth rates, indicating that gene knockout had no significant effect on cell growth. However, when cultured in medium containing 1.6 g/L CA, the growth rate of the strain *ERG6*Δ was significantly lower than that of BY4741 (Figure 2B), indicating that *ERG6* gene knockout has a significant impact on CA tolerance in cells. In this experiment, the cells from the strain *ERG6*Δ and BY4741 strain in the lag phase under CA pressure were collected for transcriptome and metabolome analysis.

As shown in Supplementary Table S1 of Supplementarymaterial Data Sheet 2, the clean bases of almost all samples are larger than 6.0G. *S. cerevisiae* has 6,000 genes, each about 1,000 bp, and one transcriptome is about 6 × 10^6^ bp, which suggests that the sequencing depth in this study is greater than 1,000×. Generally, a sequencing depth is required around 100× while depth in this study far outstrips the standard. With Q20 and Q30 of this sequencing both reaching over 90%, a low error probability and high quality of the sequencing data are guaranteed. In a word, the sufficiency and quality of the sequencing data in this study make them suitable for analysis. As shown in Supplementary Figure S1, correlation analysis was conducted between samples, and the samples from the same biological replicate were highly correlated, indicating that the data from each replicate can be used for further research and analysis. Comparing the paired pre-treatment and post-treatment sequencing results, it was found that each strain had differentially expressed genes under CA stress. The BY4741 standard strain had a total of 2063 differentially expressed genes after CA treatment, including 1,099 up-regulated genes and 964 down-regulated genes (Figure 2C). The strain *ERG6*Δ had a total of 2,349 differentially expressed genes, including 1,327 up-regulated genes and 1,022 down-regulated genes (Figure 2D).

By comparing the differentially expressed up-regulated genes between the strain *ERG6*Δ (YML008C) and the standard strain BY4741 (YOK202W) using the Venn diagram (Figure 2E), it was found that out of over 1,300 genes, the standard strain BY4741 had 217 unique genes, while the strain *ERG6*Δ had 445 unique genes with 882 genes shared. Using Cytoscape3.9.1, gene enrichment analysis was performed on 445 up-regulated genes unique to the strain *ERG6*Δ (Supplementary Table S2). The genes are mainly enriched in 8 pathways, including the intrinsic component of the membrane, organic cyclic compound metabolic process, and intrinsic part (Supplementary Table S2). By comparing the differentially expressed down-regulated genes between the strains *ERG6*Δ and BY4741 using the Venn diagram (Figure 2F), it was found that out of approximately 1,000 genes, the standard strain BY4741 has 280 unique genes, while the strain *ERG6*Δ has 338 unique genes with 684 genes shared by both. Using Cytoscape 3.9.1, gene enrichment analysis was performed on 338 down-regulated genes unique to the strain *ERG6*Δ (Supplementary Table S3). The genes are mainly enriched in 14 pathways, including macropolar complex subunit organization, mRNA metabolic process, oxidoreductase activity, acting on the CH-CH group of donors, and cell cycle.

Supplementary Figures S2, S3 show the Pearson correlation analysis results between positive and negative charge metabolites in replicate samples. As can be seen from the Supplementary Figures S2, S3, there is a high correlation between the data from replicate samples in this metabolomic sequencing, indicating that the data is reliable for subsequent analysis. After CA treatment, the principal component analysis of the strains *ERG6*Δ and BY4741 in metabolomic sequencing is shown in Figures 2G,H. It can be seen that there is a large difference in the principal components of the metabolome between the strains *ERG6*Δ and BY4741, indicating that there are significant differences in metabolites between the two strains. Analysis of differential expression of metabolites in various strains under CA stress revealed that 251 and 214 metabolites were up-and down-regulated in the strain *ERG6*Δ, respectively, while 249 and 204 metabolites were up-and down-regulated in the strain BY4741, respectively. Through Venn diagram analysis, it was found that there were one and two common up-and down-regulated metabolites in the strains *ERG6*Δ and BY4741, respectively (Supplementary Figures S4, S5). In order to gain a deeper understanding of the specific expression of metabolites in the strain *ERG6*Δ, a metabolite enrichment analysis was performed. As shown in Supplementary Figures S6, S7, the analysis revealed that the metabolites specifically up-regulated in the strain *ERG6*Δ were enriched in pathways such as pentose phosphate pathway, starch and sucrose metabolism, glycolysis/gluconeogenesis, galactose metabolism, beta-alanine metabolism, purine metabolism, amino sugar and nucleotide sugar metabolism (Supplementary Figure S6) while the metabolites specifically down-regulated in the strain *ERG6*Δ were enriched in pathways such as pyrimidine metabolism, glutathione metabolism, arginine and proline metabolism, purine metabolism, arginine biosynthesis, and so on (Supplementary Figure S7).

### 3.3 Stress response mediated by cell wall-membrane system

**Figure 3:**
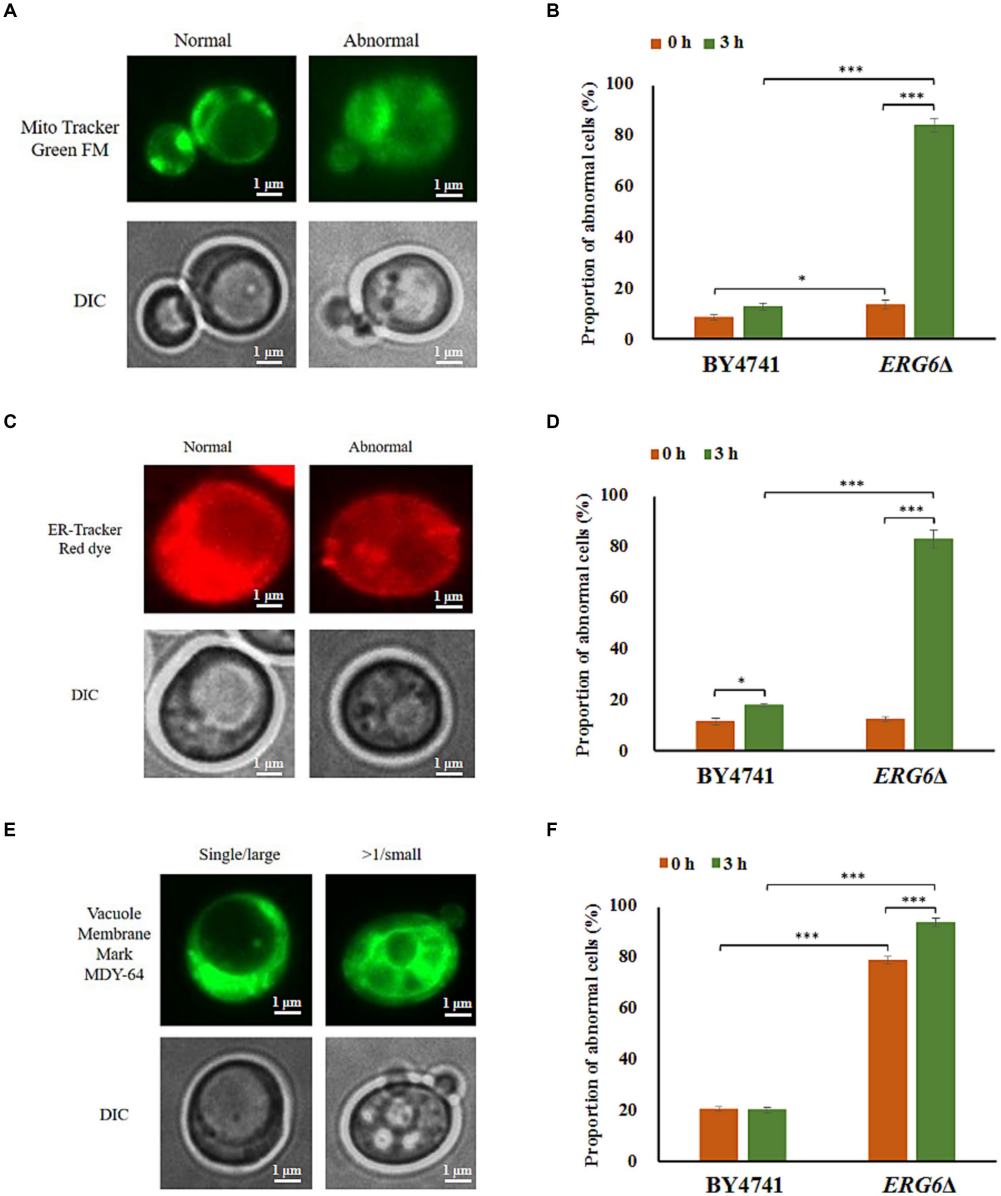
Morphological changes of mitochondria (MT), endoplasmic reticulum (ER), and vacuoles (VC) in the strain BY4741 and *ERG6*Δ under CA stress. Different morphologies of MT **(A)**, ER **(C)**, VC **(E)** in cells. The proportion of cells that displayed abnormal MT **(B)**, abnormal ER **(D)**, and more than single small VC **(F)** after CA treatment for 0 and 3 h. Mito Tracker Green FM: the mitochondria-specific dye. ER-Tracker Red dye: Endoplasmic reticulum stain. Vacuole Membrane Marker MDY-64: vacuole dyeing agent. Single/large: single large vacuole. > 1/ Small: more than single small vacuole. DIC: differential interference microscope. *p < 0.05, ***p < 0.001 indicates significant differences. The data represent averages of three experiments. *p*-values using student’s *t*-test. At least 100 cells were examined on each bright-field image.

As *ERG6* is a key gene for sterol synthesis, the knockout of *ERG6* has a significant impact on the synthesis of sterols. In addition, sterols are key components of the cell membrane (Frallicciardi et al., 2022), and the knockout of *ERG6* indirectly affects the membrane system. In order to clarify the impact of *ERG6* knockout on the membrane system, this experiment assessed the damage to mitochondria, endoplasmic reticulum, and vacuoles with membrane structures under CA stress (Figure 3). As shown in Figures 3A,C,E, under CA stress, mitochondria and endoplasmic reticulum (ER) exhibit normal and abnormal morphology, and cells also contain single large vacuoles and multiple large vacuoles. Abnormal mitochondria, and abnormal ER as well as multiple large vacuoles appear when mitochondria, ER, and vacuoles are damaged. As shown in Figure 3B, after 3 h of treatment, the percentage of cells with impaired mitochondria in the standard strain BY4741 increased from 8.39 to 13.38%, while the percentage of cells with impaired mitochondria in the strain *ERG6*Δ increased from 12.69 to 84.35%. In addition, after 3 h of treatment, the percentage of cells with ER damage in the standard strain BY4741 increased from 11.30 to 17.83%, while the percentage of cells with ER damage in the strain *ERG6*Δ increased from 12.38 to 83.25%. The investigation of vacuole damage revealed that the percentage of vacuole-damaged cells in the standard strain BY4741 remain almost the same before and after treatment, slightly down from 20.59 to 20.13%, whereas the percentage of vacuole-damaged cells in the strain *ERG6*Δ increased from 79.29 to 94.08%. From the above results, it can be seen that mitochondria, ER, and vacuoles with membrane structures in the strain *ERG6*Δ all suffer significant damage under CA stress. In addition, the knockout of *ERG6* causes vacuole damage in cells in the absence of CA.

**Figure 4:**
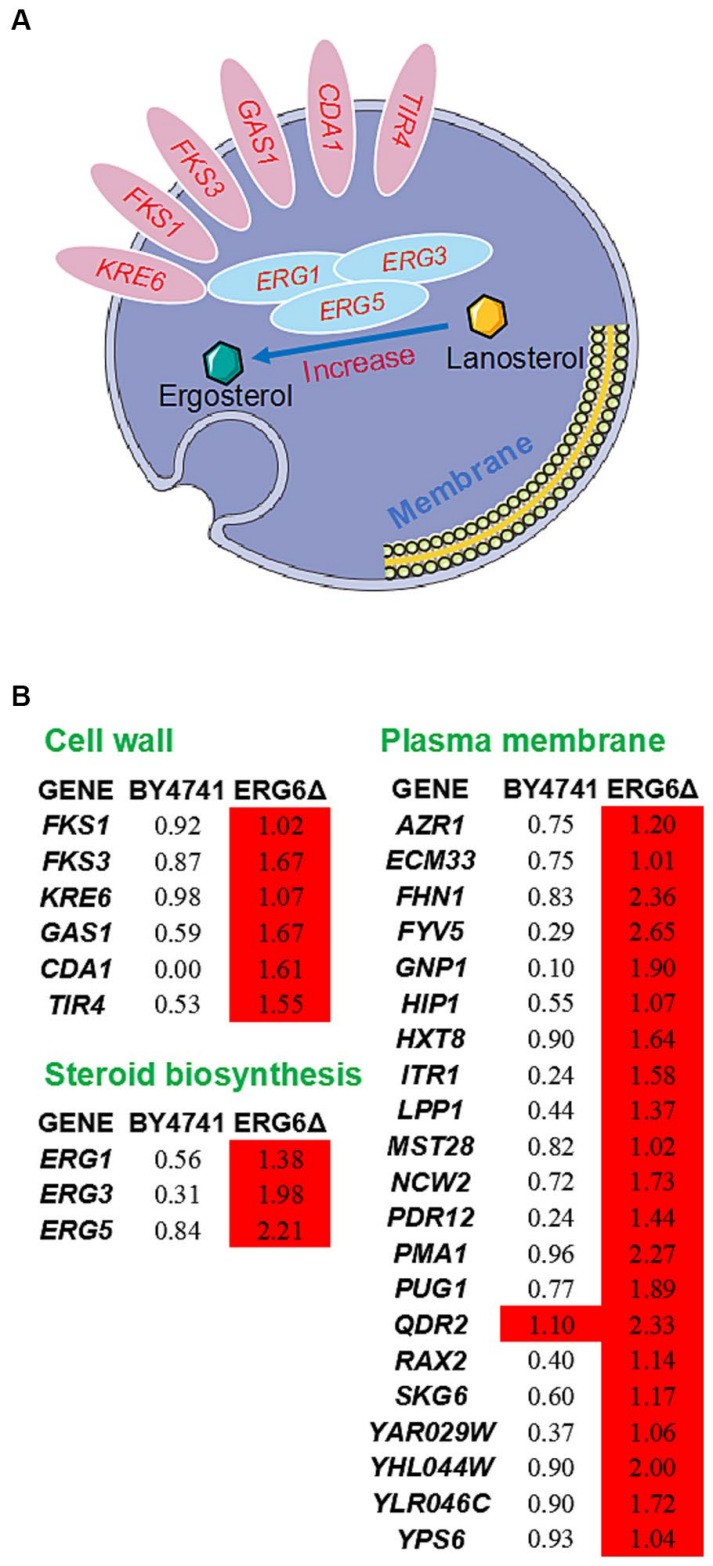
Response mechanism of cell wall-membrane system to CA in the strain *ERG6*Δ. **(A)** Schematic diagram showing the up-regulated genes related to cell wall and steroid biosynthesis in the strain *ERG6*Δ caused by CA. **(B)** The expression levels of genes with values of log2(fold change) in response to 1.6 g/L CA in the strain BY4741 and *ERG6*Δ. Red means that the gene is significantly upregulated.

Furthermore, analysis of the transcriptome sequencing data revealed that the up-regulated genes specific to the strain *ERG6*Δ were enriched in the intrinsic component of the membrane pathway (Supplementary Table S2). Through the enrichment and sorting of up-regulated genes, it was found that the genes responsible for glucan synthesis and processing/remodeling, such as *FKS1*, *FKS3*, *KRE6*, *GAS1*, the genes involved in chitin synthesis, such as *CDA1*, and the genes responsible for protein mannosylation, such as *TIR4*, all showed more than 2-fold specific up-regulation in the strain *ERG6*Δ (Figure 4). These genes are all key genes involved in the synthesis of cell wall components, and their up-regulation is likely to promote the enhancement of cell wall resistance to CA. In addition, as *ERG6* is a key gene in the sterol synthesis pathway, other genes related to sterol synthesis, such as *ERG1*, *ERG3*, and *ERG5*, were also investigated in this experiment. In the strain *ERG6*Δ, these three genes showed 2.60, 3.94, and 4.63-fold specific up-regulation under CA stress, respectively, while no such up-regulation was observed in the BY4741 strain (Figure 4). It is possible that these genes were up-regulated to compensate for the lack of sterol synthesis. Since *ERG6* has a significant impact on the membrane system, the expression levels of membrane-related genes were also analyzed in this experiment. As shown in Figure 4B, 20 membrane-related genes in the strain *ERG6*Δ showed more than 2-fold up-regulation, while no such up-regulation was observed in the strain BY4741 (Figure 4). It is also noteworthy that though up-regulated in both strains, the cation transport-related gene *QDR2* showed much stronger up-regulation in the strain *ERG6*Δ (5.03-fold) than the that in the strain BY4741(2.14-fold) (Figure 4B). It is reasonable to assume that the knockout of *ERG6* leads to a decrease in sterol content, which is likely to activate the resistance of the cell wall and membrane system to CA.

### 3.4 *ERG6* knockout promotes rearrangement of the glutathione metabolism pathway

**Figure 5:**
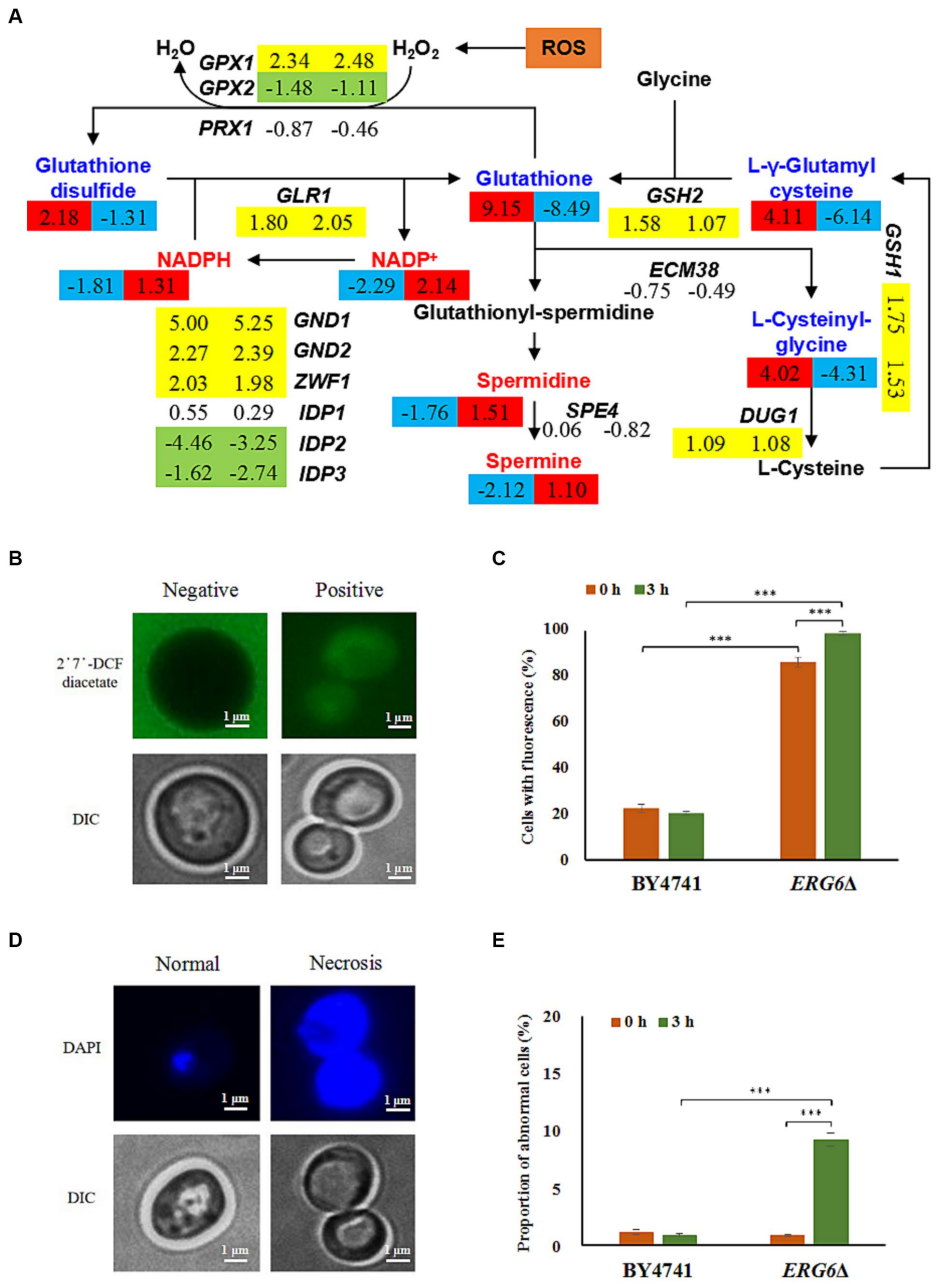
Glutathione-mediated scavenging of reactive oxygen species (ROS) and ROS-induced chromatin damage in the strain BY4741 and *ERG6*Δ under CA stress. **(A)** Changes in the expression levels of key genes and the content of key metabolites in glutathione metabolism pathway. Contents of metabolites and expression of genes with values of log2(fold change) in the strain BY4741 (left column) and *ERG6*Δ (right column) after 1.6 g/L CA treatment for 3 h, compared to the treatment for 0 h. Yellow means that the gene is significantly up-regulated. Green means that the gene is significantly down-regulated. Red means that the content of metabolites is significantly increased. Blue means that the content of metabolites is significantly decreased. Accumulation of ROS **(B)** and different morphologies of chromatin **(D)** in cells. The proportion of cells that contained ROS **(C)** and displayed necrosis **(E)** in the strain BY4741 and *ERG6*Δ after CA treatment for 0 and 3 h. 2′7′-DCF diacetate: ROS indicator dye. DAPI: DNA specific dye diamino phenylindole. ***p < 0.001 indicates significant differences. The data represent averages of three experiments. *p*-values using student’s *t*-test. At least 100 cells were examined on each bright-field image.

The metabolic analysis of the strain *ERG6*Δ and BY4741 exposed to CA revealed that the metabolites specifically down-regulated in the strain *ERG6*Δ were enriched in the glutathione metabolism pathway. By analyzing the expression levels of metabolites and key genes in this pathway, it was found that the glutathione, glutathione disulfide, and glutathione synthesis-related metabolites L-γ-glutamylcysteine, L-Cysteinyl-glycine showed a 359.54, 2.48, 70.52, and 19.84-fold decrease, respectively, in the strain *ERG6*Δ, while in the strain BY4741 they showed a 568.10, 4.53, 17.27, and 16.22-fold increase, respectively. This indicates that the knockout of *ERG6* significantly affected the synthesis of glutathione (Figure 5A). Is this phenomenon caused by changes in the expression levels of genes that control this pathway? The investigation showed that the expression levels of the genes *GPX1*, *GPX2*, *PRX1*, *GLR1*, *GSH2*, *GSH1*, *DUG1*, and *ECM38* involved in the synthesis of metabolites in the strain *ERG6*Δ and BY4741 showed the same trend under CA stress, such as gene *GLR1*, which showed a 3.48-and 4.14-fold increase in the two strains, respectively (Figure 5A). This suggests that the changes in glutathione and its synthesis-related metabolites are not caused by the changes in the transcription levels of the above genes. In the reaction catalyzed by Prx1p, Gpx1p, and Gpx2p with glutathione as the substrate, H_2_O_2_ in the ROS can be converted to H_2_O, which can greatly reduce the oxidative stress caused by accumulation of ROS in cells. However, the expression levels of the above three genes did not show significant differences between the two strains, so these three genes cannot play a promoting role in the clearance of ROS. From Figures 5B,C, it can be seen that the percentages of cells containing ROS in the strain BY4741 after CA treatment for 0 h and 3 h stayed at 22.49 and 20.30%, respectively, indicating that CA does not stimulate the accumulation of intracellular ROS in the strain BY4741. It is speculated that glutathione, which is up-regulated by 359.54-fold, may promote the effective clearance of intracellular ROS in the strain BY4741 (Figure 5A). However, the percentage of cells containing ROS in the strain *ERG6*Δ before treatment with CA was 86.36% and after 3 h of treatment it was further increased to 98.83%, which indicates that knocking out *ERG6* promotes intracellular ROS accumulation whether in the presence or absence of CA. The above indicates that the clearance efficiency of ROS is significantly reduced in the strain *ERG6*Δ, which is probably related to the decrease in glutathione (Figure 5A). Previously, it was found that excessive accumulation of intracellular ROS can cause damage to intracellular nucleic acids (Nakamura and Takada, 2021). Therefore, this experiment also investigated the chromatin damage in the strain *ERG6*Δ and BY4741 (Figures 5D,E). From Figure 5E, it can be seen that the necrosis ratio in the two strains was 1.17 and 0.89% at 0 h after CA treatment, but the necrosis ratio in the strain *ERG6*Δ reached 9.21% after 3 h of CA treatment, while there was no significant change in the necrosis ratio in the strain BY4741. This indicates that CA can damage the chromatin in the strain *ERG6*Δ, which is likely related to the accumulation of ROS.

Although the glutathione content in the strain *ERG6*Δ significantly decreased under CA stress, the synthesis of spermidine and spermine, precursors for glutathione, significantly increased by 2.85 and 2.14 times, respectively (Figure 5A). In contrast, the levels of these two metabolites decreased by 3.39 and 4.35 times in the strain BY4741 under CA stress. This indicates that CA stress promotes the accumulation of spermidine and spermine in the strain *ERG6*Δ. Furthermore, this experiment also found that the coenzymes NADPH and NADP+, which catalyze the formation of glutathione from glutathione disulfide, increased by 2.48 and 4.41 times, respectively, in the strain *ERG6*Δ, but decreased by 3.51 and 4.89 times in strain BY4741 under CA stress (Figure 5A). Analysis of the expression levels of genes involved in the synthesis of NADPH and NADP^+^ in the glutathione metabolism pathway showed that *GLR1*, *GND1*, *GND2*, *ZWF1*, *IDP1*, *IDP2*, *IDP3* showed the same trend in the expression levels of the strain *ERG6*Δ and BY4741 after CA treatment for 3 h (Figure 5A). Therefore, the changes in the expression levels of genes involved in the synthesis of NADPH and NADP^+^ did not directly affect the levels of NADPH and NADP^+^ in the two strains. The decrease in glutathione and glutathione disulfide level and the increase in NADPH and NADP^+^ levels lead to a hypothesis that other synthetic pathways promote the large-scale synthesis of NADPH and NADP^+^.

### 3.5 Pentose phosphate pathway rearrangement promotes NADPH synthesis

**Figure 6:**
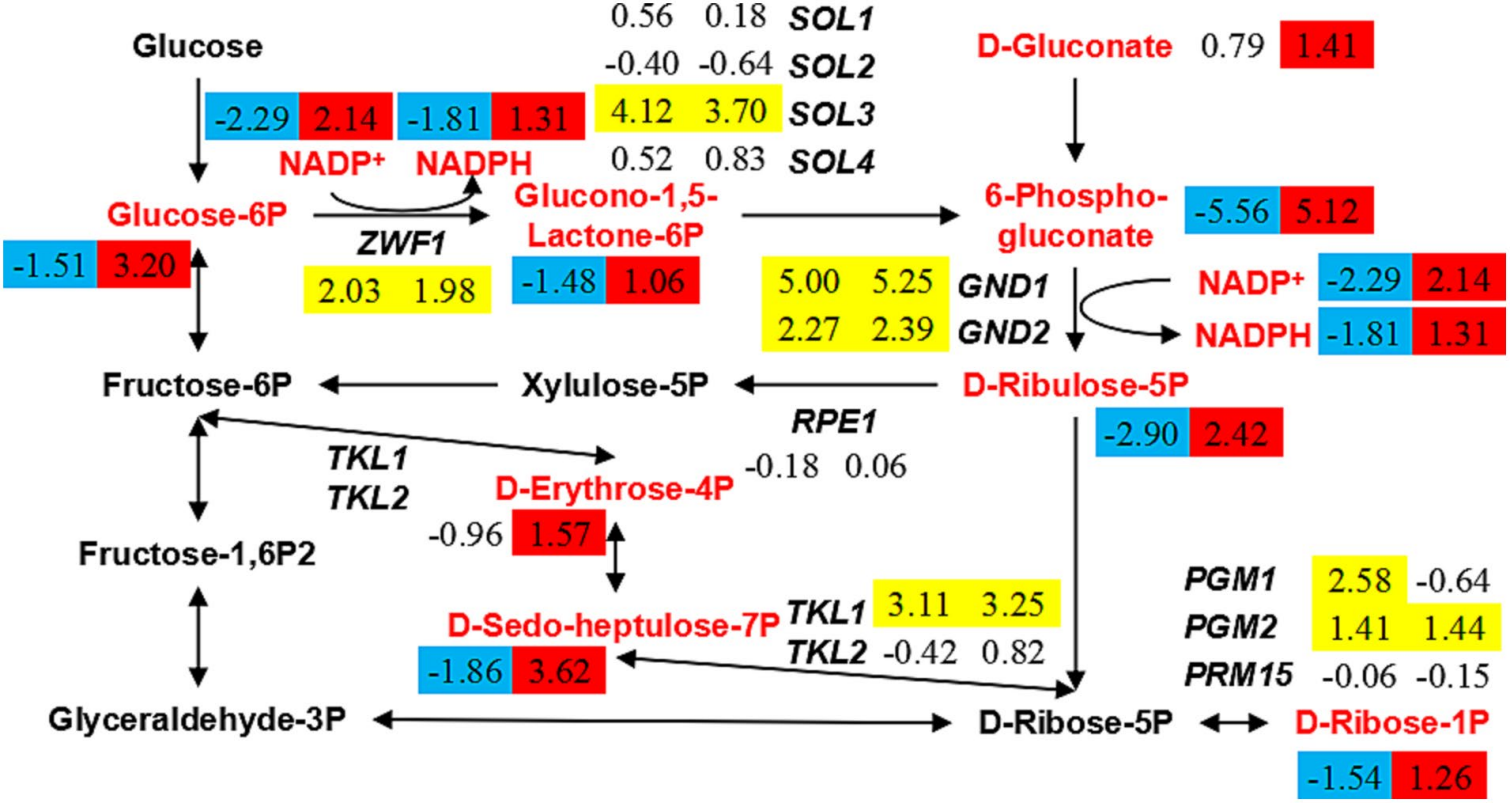
Changes in the expression levels of key genes and the content of key metabolites in pentose phosphate pathway in the strain BY4741 and *ERG6*Δ after 1.6 g/L CA treatment. Contents of metabolites and expression of genes with values of log2(fold change) in the strain BY4741 (left column) and *ERG6*Δ (right column) after 1.6 g/L CA treatment for 3 h, compared to the treatment for 0 h. Yellow means that the gene is significantly up-regulated. Red means that the content of metabolites is significantly increased. Blue means that the content of metabolites is significantly decreased.

According to the previous results, the low level of glutathione metabolism in the strain *ERG6*Δ under CA stress cannot promote or even hinder the synthesis of NADPH and NADP^+^. In order to clarify the reason for the increase in NADPH and NADP^+^ content, this experiment investigated the metabolic pathways involved in the synthesis of NADPH and NADP^+^. The pentose phosphate pathway (PPP) is a key pathway specifically up-regulated in the strain *ERG6*Δ under CA stress, and it is speculated that the specific up-regulation of key metabolites in this pathway affects the synthesis of NADPH and NADP^+^. In the PPP, the contents of glucose-6-phosphate, glucono-1,5-lactone-6-phosphate, 6-phospho-gluconate, D-ribulose-5-phosphate, D-gluconate, D-erythrose-4-phosphate, and D-sedo-heptulose-7-phosphate increased more than 2-fold in the strain *ERG6*Δ under CA stress, while in the strain BY4741 they decreased more than 2-fold (Figure 6). Among them, the metabolites glucose-6-phosphate, glucono-1,5-lactone-6-phosphate, 6-phospho-gluconate, and D-ribulose-5-phosphate, which are involved in the synthesis of NADPH and NADP^+^, increased by 9.19, 2.08, 34.78, and 5.35 times, respectively, in the strain *ERG6*Δ (Figure 6). In addition, the key enzyme genes *ZWF1*, *GND1*, and *GND2*, which catalyze these two reactions, showed the same trend in the expression levels of both strains under CA stress (Figure 6). Based on the above results, it can be concluded that the increase in metabolites glucose-6-phosphate, glucono-1,5-lactone-6-phosphate, 6-phospho-gluconate, and D-ribulose-5-phosphate in the PPP is an important reason for the increase in NADPH and NADP^+^. Besides, analysis of the transcription levels of enzyme genes involved in metabolite synthesis revealed that *SOL1*, *SOL2*, *SOL3*, *SOL4*, *RPE1*, *TKL1*, *TKL2*, and other genes showed the same trend in the expression levels of the two strains under CA stress (Figure 6). For example, *SOL3* was up-regulated by 17.38 and 12.99 times in the strain BY4741 and *ERG6*Δ, respectively, while the expression levels of *SOL1*, *SOL2*, *SOL4*, and *RPE1* did not change significantly in either strain (Figure 6). The above results suggest that the increase in the content of key metabolites in the PPP pathway is not triggered by changes in the transcription levels of key enzyme genes. It can therefore be concluded that *ERG6* knockout can promote an increase in the content of key metabolites in the PPP pathway under CA stress.

## 4 Discussion

Enhancing the resistance of engineered *S. cerevisiae* to CA treatment is the key to promoting the production of muconate from CA, a raw material for fermentation. Previous studies have found that the *YAP1* and *SKN7* knockout strains exhibit significant sensitivity to CA treatment. Overexpression of *YAP1* increases the resistance of yeast cells to CA treatment (North et al., 2011), but there has been no study on the mechanism of CA resistance tolerance and the influence of non-essential genes on CA tolerance.

In previous studies, BY4743 has used as a background knockout strain library to conduct sensitivity analysis of knockout strains in the CA environment, and it was found that oxidative stress, membrane phospholipids, endoplasmic reticulum, and vacuoles are all related to CA resistance (North et al., 2011). In this experiment, however, with BY4741 used as a background knockout strain, sensitivity analysis revealed that genes related to CA tolerance were mainly enriched in the ethanol metabolism pathway (*IRE1*, *ERG2*, *ERG6*, *UME6*) and the mitochondrial pathway (*IML1*, *MMM1*, *PRO41*). The discoveries of the two pathways related to CA tolerance shed light on the underlying mechanism of CA resistance.

Although *ERG2*, *ERG6*, *UME6*, and *IRE1* are all related to ethanol metabolism, why are these knockout strains sensitive to CA stress? It is possible that the knockout of these genes leads to a decrease in ethanol metabolism efficiency, resulting in ethanol accumulation within the cell. Previous studies have shown that ethanol accumulation can increase membrane fluidity, leading to the leakage of cofactors and the inactivation of membrane enzymes and cytosolic enzymes (Huffer et al., 2011). Under non-CA stress, the knockout strains of these genes did not show significant growth inhibition. It was speculated that the ethanol accumulation caused by these gene knockouts was not sufficient to cause growth inhibition. Catechol has oxidative-reductive activity and can induce further membrane damage. Additionally, after membrane damage, CA can more easily enter cells and cause genetic toxicity such as DNA and protein damage (Vazhappilly et al., 2021). Therefore, these may be the reasons why *ERG2* and *ERG6* knockout strains are extremely sensitive to CA stress. *ERG2* encodes a C-8 sterol isomerase, which catalyzes the isomerization of the delta-8 double bond to the delta-7 position in the intermediate step of ergosterol biosynthesis. *ERG6* encodes delta (24)-sterol C-methyltransferase, which converts zymosterol to fecosterol by methylating C-24 in the ergosterol biosynthetic pathway (Jordá and Puig, 2020). Ergosterol is an essential component of fungal cell membranes, which determines the fluidity, permeability, and activity of membrane-associated proteins (Jordá and Puig, 2020). It was found in this experiment that the knockout of *ERG2* and *ERG6* caused the yeast strain to become sentitive to CA stress. Given that deletion of *ERG2* and *ERG6* increased sensitivity of the strain to fluconazole butylbenzimidazole, which contains a phenyl ring (Bhattacharya et al., 2018), it can be speculated that *ERG2* and *ERG6* may be related to resistance to compounds with phenyl rings. The knockout of *ERG6* can lead to the accumulation of cholesta-type sterols (including zymosterol) and increase gene expression of the final step of the ergosterol biosynthesis pathway (*ERG2*, *ERG3*, *ERG5*, *ERG4*) (Konecna et al., 2016; Ahmad et al., 2019). In this study, the metabolome analysis detected no accumulation of zymosterol and zymosterone, but it was detected that the ergosterol content in strains BY4741 and *ERG6*Δ increased by 0.46 times and decreased by 0.65 times under CA stress, respectively. This indicates that the knockout of *ERG6* can lower ergosterol levels. The investigation of gene expression of sterol synthesis pathway in the two strains under CA stress showed that the genes encoding enzymes that catalyze the synthesis of zymosterol and zymosterone, *ERG26* and *ERG27*, as well *as ERG2* and *ERG4*, were more than 2-fold up-regulated. Moreover, under CA stress, the expression of *ERG3* and *ERG5* was down-regulated by more than 2 times in the strain *ERG6*Δ. Specific upregulation occurred in the strain *ERG6*Δ. The results of this study are consistent with previous research. This suggests that when *ERG6* is knocked out, other genes on the pathway are overexpressed to compensate for the lack of ergosterol synthesis.

In view of the results of the omics analysis, it was found that the knockout of *ERG6* caused the upregulation of genes related to cell wall-membrane system under CA stress. However, there was no significant upregulation of membrane system and cell wall genes in the strain *ERG6*Δ without CA stress. Therefore, it can be inferred that the knockout of *ERG6* cannot affect the expression levels of these cell wall-membrane system related genes, but rather is caused by CA. In the analysis of apoptosis of human red blood cells under CA stress, it was found that CA can increase an increase permeability of cell membrane (Bukowska et al., 2015). Given the above findings, it can be speculated that the *ERG6*-regulated ergosterol impairs membrane function in the strain *ERG6*Δ and CA further increases membrane permeability, causing stress on the cell wall-membrane system, which together induce the upregulation of related gene expression. However, it is also possible that these genes interact with *ERG6*, and when *ERG6* is knocked out, these genes are also upregulated. Due to the lack of research on the interaction between, an interaction analysis was conducted of up-regulated membrane genes in the strain *ERG6*Δ (Supplementary Table S2) using TheCellMap.^2^ As shown in Supplementary Figure S8, among these genes only *ERG3* and *ERG5* are directly related to *ERG6*. In addition, the interaction analysis was conducted between down-regulated genes *TIR4*, *CDA1*, *GAS1*, *FKS3*, *FKS1*, *KRE6* in the cell wall and sterol synthesis-related genes *ERG1*, *ERG3*, *ERG5* and *ERG6* (Figure 4B), in which only *ERG3* and *ERG5* were found to have a direct relationship with *ERG6* (Supplementary Figure S9). Therefore, it can be speculated that due to the direct correlation between three genes, *ERG3* and *ERG5* are specificly upregulated in the strain *ERG6*Δ in response to CA stress, leading to a decrease in ergosterol level. In addition to the ergosterol biosynthesis pathway, there is also a cholesterol synthesis pathway, both steroid synthesis pathways (Morikawa et al., 2006; Wriessnegger and Pichler, 2013). Due to the specific upregulation of the key gene *ERG3* responsible for the cholesterol pathway in the strain *ERG6*Δ under CA stress, other synthesis-related genes, such as *TGL1*, *TGL5*, *ARE1*, and *ARE2*, showed no significant differences between the two strains under CA stress. Therefore, it can be speculated that the concentration of cholesterol may have increased to some extent in the strain *ERG6*Δ under CA stress. Cholesterol is a key component of cell membranes, and the increase in cholesterol content can enhance the stability and tolerance of cell membranes (Ikonen and Zhou, 2021). In conclusion, the knockout of *ERG6* may possibly affect the synthesis of ergosterol, but it may also activate the stress response of yeast to CA mediated by cholesterol.

The final step of sterol production in *S. cerevisiae* occurs in the endoplasmic reticulum (ER). Afterwards, ergosterol molecules are transferred as sterol esters (free sterols) to various subcellular compartments, such as mitochondria, vacuoles, and the plasma membrane and ER membrane system. In fungi, sterols are closely associated with unsaturated fatty acids and play an important role in maintaining the microfluidic state of the membrane (Oliveira et al., 2020). Observation of the subcellular structure revealed that the vacuolar suffer severe structural damage in the strain *ERG6*Δ under non-CA stres. It is possible that *ERG6* is involved in the microfluidic state of vacuoles and lipid autophagy in vacuoles, and the deletion of it has caused severe damage to vacuoles under non-CA stress (Zhang et al., 2020). However, under non-CA stress, mitochondria and ER in the strain *ERG6*Δ function well to maintain homeostasis and support normal cell growth. This suggests that free sterols play different roles in mitochondria, endoplasmic reticulum, and vacuoles, which needs further study. However, exposure to CA stress exterted a considerable damage on the mitochondria, endoplasmic reticulum, and vacuoles in the strain *ERG6*Δ. Research has proved that ergosterol plays an important role in maintaining mitochondrial DNA on the inner mitochondrial membrane (Cirigliano et al., 2019). Other studies have reported that sterol metabolism is also linked to the biosynthesis of mitochondrial (Fe-S) clusters (Ward et al., 2018). Functional mitochondria play a crucial role in regulating yeast physiology, including energy production, lipid and cell wall biosynthesis, and iron homeostasis (Bhatia-Kiššová and Camougrand, 2010). In addition, the interaction between ergosterol and Sey1p can promote atlasin-mediated endoplasmic reticulum membrane fusion in *S. cerevisiae* (Lee et al., 2019). It can be speculated that the reduction in ergosterol content caused by the knockout of *ERG6* may have an adverse effect on the stability of mitochondria and endoplasmic reticulum, which in turn leads to an increased damage to mitochondria and endoplasmic reticulum under CA stress. Apart from this, the entry of CA into the cell as a phenolic substance (such as phenol) is presumably likely to cause damage to cell wall plasma membrane and cellular organelles (Wang H. et al., 2020). Previous studies have found that some gene knockouts do not produce noticeable accumulation of reactive oxygen species in cells under normal growth conditions (Zhao et al., 2020). However, a large amount of reactive oxygen species was observed accumulated in the strain *ERG6*Δ under non-CA stress. Research has shown that some key enzymes (Erg11p, Erg5p, Erg25p, Erg3p) in the late stage of the ergosterol biosynthesis pathway require iron as a cofactor for oxidation–reduction (da Costa et al., 2018). When the flux of the ergosterol synthesis pathway decreases, intracellular iron accumulates improperly, which trigger ROS accuculation in cells through the Fenton or Haber-Weiss reactions. Toxic to cells, excess iron replaces copper and zinc in metalloproteins, thereby altering their enzymatic activity (Elias et al., 2023). In summary, the accumulation of ROS in the strain *ERG6*Δ under non-CA stress may be caused by excessive accumulation of iron. In fact, the augmented ROS levels can be sensed and restrictively controlled by the ROS-scavenging system (Wang H. et al., 2020). However, the investigation found that the expression of active oxygen scavenging enzymes genes *SOD1*, *SOD2*, *CTT1*, *GPX1*, *GPX2* in the strain *ERG6*Δ and BY4741 did not differ significantly. In addition, glutathione is a key metabolite involved in the removal of ROS, but the glutathione level in this study showed a significant decline. It has been found that the glutathione level in *Candida tropicalis* was significantly increased under phenolic stress conditions, suggesting that glutathione is a key metabolite for improving resistance to phenolic compounds (Wang H. et al., 2020). However, in this study, under CA stress, the reduction in glutathione content in the strain *ERG6*Δ resulted in a large accumulation of ROS, which is likely an important reason for the weakening of CA resistance in *S. cerevisiae*. The above results suggest that the iron overload caused by the knockout of *ERG6* and the failure of ROS scavenging mechanisms led to the accumulation of ROS in the strain *ERG6*Δ. It is interesting to note that under CA stress, although the contents of glutathione and oxidized glutathione in the strain *ERG6*Δ decreased significantly, from which the synthesis of spermidine and spermine increased. Previous studies have found that the addition of spermidine is a key factor in improving the tolerance of *S. cerevisiae* to lignocellulose hydrolysates such as furfural, 5-hydroxymethyl-furfural, acetic acid, and phenolic compounds (Kim et al., 2015). Therefore, the accumulation of spermidine in the strain *ERG6*Δ may be of some importance in improving CA tolerance.

This experiment has unveiled for the first time the accumulation of NADPH and NADP^+^ levels in the strain *ERG6*Δ. Decreased levels of NADPH and NADP^+^ lead to decreased resistance of yeast to furfural, acetic acid, and oxidative stress (Qiu et al., 2019). In addition, it has been found that the increase of NADPH and NADP^+^ promotes the synthesis of glutathione, thereby reducing ROS levels and increasing tolerance to isobutanol and oxidants (Yoshikawa et al., 2021). Therefore, it can be concluded that the CA stress activates the NADPH and NADP^+^-mediated oxidative stress mechanism in the strain *ERG6*Δ. The study also found that the knockout of *GND1* makes yeast senstiive to isobutanol, but the NADPH and NADP^+^ levels in the *GND1* knockout strain are similiar to those in the wild-type strain (Kuroda et al., 2019). This indicates that in addition to changes in NADPH and NADP^+^ levels in the PPP pathway, there exist other factors affecting the tolerance of strains to abiotic stress. According to data analysis, the knockout of *GND1* causes changes in NADPH and NADP^+^ levels as well as changes in metabolite levels in the PPP pathway. It is therefore speculated that the flux of the PPP pathway may play a role in affecting the resistance of *S. cerevisiae* to isobutanol (Kuroda et al., 2019). In this study, the flux of most metabolites was found to increase significantly in the PPP pathway of the strain *ERG6*Δ under CA stress. Furthermore, the knockout of *RPE1* and *TKL1* genes, which are not involved in the synthesis of NADPH and NADP^+^ in the PPP pathway, also leads to increased sensitivity of *S. cerevisiae* to isobutanol stress (Kuroda et al., 2019). Rapid changes in the PPP pathway and increased flux in *E. coli* are discovered to increase the cell’s resistance to oxidative stress sensitivity (Christodoulou et al., 2018). In general, not only the increase in NADPH but also the increase in the levels of key products in the PPP synthesis pathway improve the tolerance of the strain *ERG6*Δ to CA stress.

The findings of this study have revealed the stress response and molecular mechanisms of yeast tolerance to CA. The accumulation of glutathione, ergosterol, spermidine, NADPH, and metabolites in the PPP pathway are key factors in improving CA resistance in *S. cerevisiae*. This knowledge lays the foundation for creating CA-tolerant strains and enhancing muconate production through fermentation.

## Data availability statement

The datasets presented in this study can be found in online repositories. The names of the repository/repositories and accession number(s) can be found in the article/Supplementary material.

## Author contributions

HL: Conceptualization, Data curation, Formal analysis, Investigation, Methodology, Project administration, Software, Validation, Writing – original draft, Writing – review & editing. QL: Conceptualization, Data curation, Investigation, Methodology, Validation, Writing – original draft, Writing – review & editing. YC: Conceptualization, Data curation, Investigation, Methodology, Validation, Writing – original draft, Writing – review & editing. JT: Conceptualization, Data curation, Investigation, Validation, Writing – original draft, Writing – review & editing. BM: Methodology, Writing – review & editing. FL: Methodology, Writing – review & editing. PF: Methodology, Writing – review & editing. WLi: Data curation, Writing – review & editing. JL: Data curation, Writing – review & editing. CF: Data curation, Formal analysis, Writing – review & editing. WLo: Data curation, Formal analysis, Writing – review & editing. XX: Data curation, Formal analysis, Writing – review & editing. XH: Data curation, Formal analysis, Writing – review & editing. WX: Methodology, Writing – review & editing. FY: Methodology, Writing – review & editing. MM: Writing – review & editing. BL: Supervision, Writing – review & editing. YY: Supervision, Writing – review & editing, Funding acquisition, Project administration. HW: Supervision, Writing – review & editing, Funding acquisition, Project administration.

## Footnotes

^1^https://pathways.embl.de/

^2^https://thecellmap.org/
