# Supplementary material for: Genome-wide identification of resistance genes and response mechanism analysis of key gene knockout strain to catechol in Saccharomyces cerevisiae
Source: Front Microbiol. 2024 Feb 21;15:1364425. doi: 10.3389/fmicb.2024.1364425 (PMC10915035; doi:10.3389/fmicb.2024.1364425)
Supplement: Supplementary file 2 [file Data_Sheet_1.docx]

**RNA-seq- Materials and Methods**

# Sample collection and preparation

## RNA quantification and qualification

Total amounts and integrity of RNA were assessed using the RNA Nano 6000 Assay Kit of the Bioanalyzer 2100 system (Agilent Technologies, CA, USA). (Refer to RNA quality inspection report for specific instruments)

## Library preparation for Transcriptome sequencing

Total RNA was used as input material for the RNA sample preparations. Briefly, mRNA was purified from total RNA by using poly-T oligo-attached magnetic beads. Fragmentation was carried out using divalent cations under elevated temperature in First Strand Synthesis Reaction Buffer (5X). First strand cDNA was synthesized using random hexamer primer and M-MuLV Reverse Transcriptase, then use RNaseH to degrade the RNA. Second strand cDNA synthesis was subsequently performed using DNA Polymerase I and dNTP. Remaining overhangs were converted into blunt ends via exonuclease/polymerase activities. After adenylation of 3’ ends of DNA fragments, Adaptor with hairpin loop structure were ligated to prepare for hybridization. In order to select cDNA fragments of preferentially 370~420 bp in length, the library fragments were purified with AMPure XP system (Beckman Coulter, Beverly, USA). Then PCR amplification, the PCR product was purified by AMPure XP beads, and the library was finally obtained.

In order to ensure the quality of the library, the library needs to be tested. After the construction of the library, the library was initially quantified by Qubit 2.0 Fluorometer, then diluted to 1.5 ng/μL, and the insert size of the library is detected by Agilent 2100 bioanalyzer. After insert size meets the expectation, qRT-PCR is used to accurately quantify the effective concentration of the library (the effective concentration of the library is higher than that of 2 nM) to ensure the quality of the library.

## Clustering and sequencing

After the library is qualified, the different libraries are pooling according to the effective concentration and the target amount of data off the machine, then being sequenced by the Illumina NovaSeq 6000. The end reading of 150 bp pairing is generated. The basic principle of sequencing is to synthesize and sequence at the same time (Sequencing by Synthesis). Four fluorescent labeled dNTP, DNA polymerase and splice primers were added to the sequenced flowcell and amplified. When the sequence cluster extends the complementary chain, each dNTP labeled by fluorescence can release the corresponding fluorescence. The sequencer captures the fluorescence signal and converts the optical signal into the sequencing peak by computer software, so as to obtain the sequence information of the fragment to be tested.

# Data Analysis

## Quality control

The image data measured by the high-throughput sequencer are converted into sequence data (reads) by CASAVA base recognition. Raw data (raw reads) of fastq format were firstly processed through in-house perl scripts. In this step, clean data (clean reads) were obtained by removing reads containing adapter, reads containing N base and low quality reads from raw data. At the same time, Q20, Q30 and GC content the clean data were calculated. All the downstream analyses were based on the clean data with high quality.

## Reads mapping to the reference genome

Reference genome and gene model annotation files were downloaded from genome website directly. Index of the reference genome was built using Hisat2 (v2.0.5) and paired-end clean reads were aligned to the reference genome using Hisat2 (v2.0.5). We selected Hisat2 as the mapping tool for that Hisat2 can generate a database of splice junctions based on the gene model annotation file and thus a better mapping result than other non-splice mapping tools.

## Novel transcripts prediction

The mapped reads of each sample were assembled by StringTie (v1.3.3b) (Mihaela Pertea.et al. 2015) in a reference-based approach. StringTie uses a novel network flow algorithm as well as an optional *de novo* assembly step to assemble and quantitate fulllength transcripts representing multiple splice variants for each gene locus.

## Quantification of gene expression level

The featureCounts v1.5.0-p3 was used to count the reads numbers mapped to each gene. And then FPKM of each gene was calculated based on the length of the gene and reads count mapped to this gene. FPKM, expected number of Fragments Per Kilobase of transcript sequence per Millions base pairs sequenced, considers the effect of sequencing depth and gene length for the reads count at the same time, and is currently the most commonly used method for estimating gene expression levels.

## Differential expression analysis

Differential expression analysis of two conditions/groups (two biological replicates per condition) was performed using the DESeq2 R package (1.20.0). DESeq2 provide statistical routines for determining differential expression in digital gene expression data using a model based on the negative binomial distribution. The resulting P-values were adjusted using the Benjamini and Hochberg’s approach for controlling the false discovery rate. padj<0.05 and |log2(foldchange)| > 1 were set as the threshold for significantly differential expression.

## GO and KEGG enrichment analysis of differentially expressed genes

Gene Ontology (GO) enrichment analysis of differentially expressed genes was implemented by the clusterProfiler R package (3.8.1), in which gene length bias was corrected. GO terms with corrected Pvalue less than 0.05 were considered significantly enriched by differential expressed genes. KEGG is a database resource for understanding high-level functions and utilities of the biological system, such as the cell, the organism and the ecosystem, from molecular-level information, especially large-scale molecular datasets generated by genome sequencing and other high-through put experimental technologies [(http://www.genome.jp/kegg/).](http://www.genome.jp/kegg/)) We used clusterProfiler R package (3.8.1) to test the statistical enrichment of differential expression genes in KEGG pathways.

**Gene Set Enrichment Analysis**

Gene Set Enrichment Analysis (GSEA) is a computational approach to determine if a pre-defined Gene Set can show a significant consistent difference between two biological states. The genes were ranked according to the degree of differential expression in the two samples, and then the predefined Gene Set were tested to see if they were enriched at the top or bottom of the list. Gene set enrichment analysis can include subtle expression changes. We use the local version of the GSEA analysis tool [http://www.broadinstitute.org/gsea/index.jsp,](http://www.broadinstitute.org/gsea/index.jsp) GO, KEGG data set were used for GSEA independently.

## SNP analysis

GATK2 (v3.8) software was used to perform SNP calling., and SnpEff (4.3q) software was used to annotate SNP.Raw vcf files were filtered with GATK standard filter method and other parameters (cluster:3; WindowSize:35; QD < 2.0 o; FS > 30.0; DP < 10.

## AS analysis

Alternative Splicing is an important mechanism for regulate the expression of genes and the variable of protein. rMATS(4.0.2) software was used to analysis the AS event.

## PPI analysis of differentially expressed genes

PPI analysis of differentially expressed genes was based on the STRING database, which known and predicted Protein-Protein Interactions.The Diamond software (0.9.13) was used to compare the target gene sequence with the selected reference protein sequence, and then the network was established according to the known interaction of the selected reference species.

## Weighted correlation network analysis

WGCNA (Weighted correlation network analysis) is a systematic biological method used to describe the gene association modes among different samples. it can be used to identify gene sets that are highly synergistic changed, and identify candidate biomarkers or therapeutic targets based on the coherence of gene sets and the correlation between gene sets and phenotypes. The R package WGCNA is a set of functions used to calculate various weighted association analysis, which can be used for network construction, gene screening, gene cluster identification, topological feature calculation, data simulation and visualization. WGCNA is suitable for multisample data. Generally, more than 15 samples are required. One input file is sample information, that is, a matrix describing the traits of the sample: the traits used for association analysis must be numeric; If it is a regional or categorical variable, it needs to be converted to a 0-1 matrix. The other is gene expression data. For transcriptome sequencing, FPKM can be used as gene expression data.

**Untargeted Metabolomics - Materials and Methods**

# Metabolites Extraction

The samples were placed in the EP tubes and resuspended with prechilled 80% methanol by well vortex. Then the samples were melted on ice and whirled for 30 s. After the sonification for 6 min, they werecentrifuged at 5,000 rpm, 4°C for 1 min. The supernatant was freeze-dried and dissolvedwith 10% methanol. Finally, the solution was injected into the LC-MS/MS system analysis.

# UHPLC-MS/MS Analysis

UHPLC-MS/MS analyses were performed using a Vanquish UHPLC system (ThermoFisher, Germany) coupled with an Orbitrap Q ExactiveTMHF mass spectrometer (Thermo Fisher, Germany) in Novogene Co., Ltd. (Beijing, China). Samples were injected onto a HypesilGoldcolumn (100×2.1 mm, 1.9μm) using a 17-min linear gradient at a flow rate of 0.2 mL/min. The eluents for the positive polarity mode were eluent A (0.1% FA in Water) and eluent B (Methanol). The eluents for the negative polarity mode were eluent A (5 mM ammonium acetate, pH 9.0) and eluent B (Methanol). The solvent gradient was set as follows: 2% B, 1.5 min; 2-85% B, 3 min; 85-100% B, 10 min; 100-2% B, 10.1 min; 2% B, 12 min. Q ExactiveTM HF mass spectrometer was operated in positive/negative polarity mode with spray voltage of 3.5 kV, capillary temperature of 320°C, sheath gas flow rate of 35 psi and aux gas flow rate of 10 L/min, S-lens RF level of 60, Aux gas heater temperature of 350°C.

# Data processing and metabolite identification

The raw data files generated by UHPLC-MS/MS were processed using the Compound Discoverer 3.1 (CD3.1, ThermoFisher) to perform peak alignment, peak picking, and quantitation for each metabolite. The main parameterswere set as follows: retention time tolerance, 0.2 minutes; actual mass tolerance, 5 ppm; signal intensity tolerance, 30%; signal/noise ratio, 3; and minimum intensity, et al. After that, peak intensities were normalized to the total spectral intensity. The normalized data was used to predict the molecular formula based on additive ions, molecular ion peaks and fragment ions. And then peaks were matched with the mzCloud (https://www. mzcloud.org/), mzVaultand MassListdatabase to obtain the accurate qualitative and relative quantitative results. Statistical analyses were performed using the statistical software R (R version R-3.4.3), Python (Python 2.7.6 version) and CentOS(CentOS release 6.6) , When data were not normally distributed, standardize according to the formula: sample raw quantitation value / (The sum of sample metabolite quantitation value / The sum of QC1 sample metabolite quantitation value ) to obtain relative peak areas; And compounds whose CVs of relative peak areas in QC samples were greater than 30% were removed, and finally the metabolites' identification and relative quantification results were obtained.

# Data Analysis

These metabolites were annotated using the KEGG database (https://www. genome.jp/kegg/pathway.html), HMDB database (https://hmdb.ca/metabolites) and LIPIDMaps database (<http://www.lipidmaps.org/>).
