# Supplementary material for: Genome-wide identification of resistance genes and response mechanism analysis of key gene knockout strain to catechol in Saccharomyces cerevisiae
Source: Front Microbiol. 2024 Feb 21;15:1364425. doi: 10.3389/fmicb.2024.1364425 (PMC10915035; doi:10.3389/fmicb.2024.1364425)
Supplement: Supplementary file 3 [file Data_Sheet_2.docx]

**Table S1.** **The transcriptome sequencing data.**

| **Sample** | **Clean reads (M)** | **Clean bases (G)** | **Q20** | **Q30** | **GC** **Content** | **Sequencing depth (×)** |
| --- | --- | --- | --- | --- | --- | --- |
| YOK202W_Q_CA_1 | 44,396,716 | 6.66 | 97.26 | 92.31 | 41.20 | 1110 |
| YOK202W_Q_CA_2 | 41,519,114 | 6.23 | 97.07 | 91.85 | 41.12 | 1038 |
| YOK202W_Q_CA_3 | 41,405,900 | 6.21 | 97.25 | 92.19 | 41.30 | 1035 |
| YML008C_Q_CA_1 | 40,772,824 | 6.12 | 97.44 | 92.66 | 41.21 | 1020 |
| YML008C_Q_CA_2 | 41,978,202 | 6.30 | 97.01 | 91.68 | 41.36 | 1050 |
| YML008C_Q_CA_3 | 46,975,738 | 7.05 | 97.50 | 92.84 | 41.38 | 1175 |
| YOK202W_H_CA_1 | 39,022,936 | 5.85 | 97.39 | 92.51 | 41.57 | 975 |
| YOK202W_H_CA_2 | 39,727,306 | 5.96 | 97.34 | 92.46 | 41.54 | 993 |
| YOK202W_H_CA_3 | 38,999,600 | 5.85 | 97.36 | 92.52 | 41.63 | 975 |
| YML008C_H_CA_1 | 40,716,072 | 6.11 | 97.32 | 92.42 | 41.46 | 1018 |
| YML008C_H_CA_2 | 42,792,606 | 6.42 | 97.41 | 92.57 | 41.56 | 1070 |
| YML008C_H_CA_3 | 40,154,916 | 6.02 | 97.50 | 92.79 | 41.54 | 1003 |

Sequencing depth=Clean bases/(Total number of genes×gene length). Total number of all the genes is about 6000 in *Saccharomyces cerevisiaes* (<https://www.ncbi.nlm.nih.gov/genome>), and average length of all the genes in *S. cerevisiae*is estimated to 1000 bp.


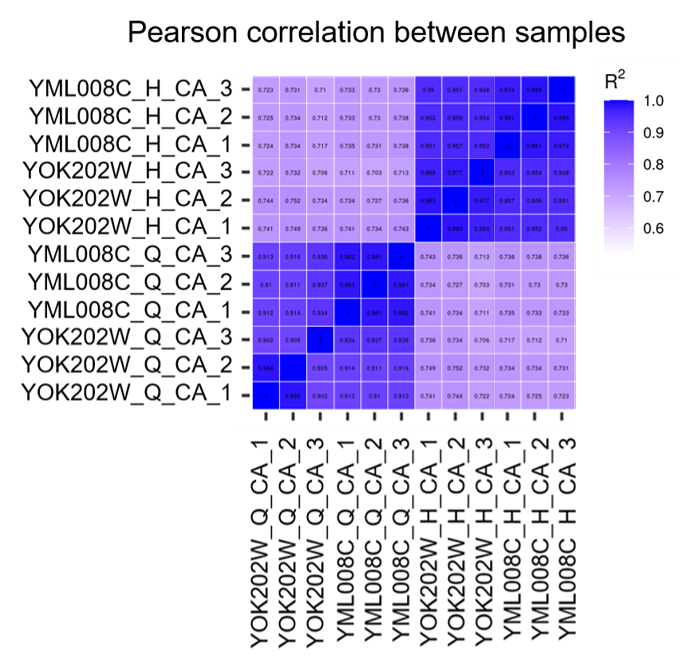


**Figure S1** **Pearson correlation between transcriptome sequencing data of the strain BY4741 and *ERG6*Δ**. YOK202W_Q_CA and YOK202W_H_CA represent the strain BY4741 treated with CA for 0 h and 3 h, respectively. YML008C_Q_CA and YML008C_H_CA represent the strain *ERG6*Δ treated with CA for 0 h and 3 h, respectively.

**Table S2. Enrichment analysis of specific up-regulated genes in the strain *ERG6*Δ.**

| ID | Term | Associated Genes Found |
| --- | --- | --- |
| GO:0031224 | Intrinsic component of membrane | *AIM34, AZR1, CCW12, CDS1, COS2, ECM12, ECM19, ECM3, ECM33, ERG1, ERG3, ERP1, FHN1, FIT1, FKS1, FKS3, FSF1, FYV5, GAS1, GNP1, GUP2, HIP1, HXT8, ICS3, ITR1, JEM1, KRE6, LPP1, MRS3, MRX11, MST28, NCW2, PAU12, PAU20, PDR12, PHS1, PIC2, PKR1, PMA1, PMT3, PMT5, PRM10, PSD1, PSY3, PUG1, QDR2, RAX2, RCE1, SBH1, SEC61, SKG6, SPR2, SSS1, STE24, SVP26, SWF1, TDA5, TIR4, TMN3, TPC1, VPH2, YAR029W, YBR196C-A, YDL211C, YDL218W, YDR089W, YHC3, YHL044W, YHR007C-A, YIL029C, YIL060W, YJL028W, YJL193W, YLR036C, YLR046C, YML018C, YMR010W, YMR244W, YNR061C, YNR065C, YOP1, YOR161C-C, YPR114W, YPS6* |
| GO:1901360 | Organic cyclic compound metabolic process | *ACM1, ADK1, ADO1, AIR1, ARG5,6, ARO7, BNA5, CCA1, CDC45, CDC9, CIN5, COA6, DAL7, DAL80, DOT6, DUT1, DXO1, ECO1, ENO1, ERG1, ERG3, ERG5, ESC8, EXO1, FKH1, FOL1, GAS1, GLK1, GRC3, GSP1, GUK1, HEM12, HEM13, HES1, HNT1, HOP2, HSL7, IME2, IOC3, IRC19, KDX1, KRE5, KTI12, MIS1, MRS1, MRS3, MSE1, MSW1, NRD1, NRG2, OGG1, ORC5, PDC6, PET54, PIF1, PMA1, PNC1, POL30, POL32, PSY3, PTI1, PUS2, QRI1, REC102, RED1, REV7, RFA2, RMA1, RMI1, RNR2, RPB11, RPS9A, RRP1, RTT109, SAE3, SLT2, SNU13, SPT21, SRL1, TAD2, TEA1, TFC8, THI11, THI22, THI4, TOF1, TOP3, TPA1, TRM9, URA6, YCS4, YMR262W, YNL247W, YPL216W, YPR127W, YPR196W, YRA1, YRF1-4, YRF1-5, ZUO1* |
| GO:0044424 | Intracellular part | *ACM1, ADK1, ADO1, ADY3, AIM34, AIR1, ALF1, ALK2, AMA1, APC1, APM3, ARG5,6, BET5, BNA5, BUB1, BUL2, CAC2, CCA1, CDC45, CDC9, CDS1, CIK1, CIN2, CIN5, CIS1, CLB4, CMR1, COA6, COG3, COQ8, COS2, CSR1, DAL7, DAL80, DGR1, DOT6, DUG3, DUO1, DXO1, ECM19, ECM25, ECM3, ECM31, ECO1, ENO1, ERG1, ERG3, ERP1, ESC8, EXO1, FKH1, FKS1, FKS3, FOL1, FPR2, FSF1, GAL7, GAS1, GEA2, GIC2, GLY1, GNP1, GRC3, GRH1, GSP1, GUK1, HEM12, HEM13, HIP1, HIS6, HOP2, HOT13, HSL7, HUA2, IME2, IML3, IOC3, IPL1, JEM1, JLP2, KDX1, KIP2, KRE5, KRE6, KTI12, LCB5, LPP1, MHT1, MIS1, MON1, MRS1, MRS3, MRX11, MSE1, MSI1, MST28, MSW1, MTW1, NMT1, NRD1, NRG2, OGG1, ORC5, PAC1, PAL1, PDC6, PET54, PHS1, PIC2, PIF1, PKR1, PMT3, PMT5, PNC1, POL30, POL32, PPZ1, PSD1, PSY3, PTH4, PTI1, PTP1, PUG1, PUS2, PXL1, PYC2, QRI1, RBL2, RCE1, REC102, RED1, REV7, RFA2, RFS1, RGA1, RHO3, RMA1, RMI1, RNP1, RNR2, RPB11, RPS9A, RRP1, RTC4, RTT109, SAE3, SBH1, SCW4, SEC24, SEC61, SGO1, SKG6, SLI15, SLT2, SNO4, SNU13, SPC98, SPH1, SPO22, SPO77, SPT21, SRM1, SRX1, SSK22, SSS1, STE23, STE24, STU2, SVL3, SVP26, SWE1, SWF1, TAD2, TDA5, TEA1, TFC8, THI4, TMN3, TOF1, TOP3, TOS3, TPA1, TPC1, TRM9, TRS31, TRS65, TUB4, TWF1, URA6, VPH2, YAR029W, YCS4, YDL211C, YDR089W, YGR066C, YHC3, YHL044W, YIH1, YIL060W, YML018C, YNL217W, YNR061C, YOP1, YPL216W, YPL245W, YPP1, YPR1, YPR114W, YPR127W, YPR196W, YRA1, YRF1-4, YRF1-5, ZUO1* |
| GO:1903047 | Mitotic cell cycle process | *ACM1, ALK2, APC1, BUB1, CDC45, CDC9, CIK1, CLB4, DUO1, ECO1, FKH1, GIC2, HSL7, IML3, IPL1, KDX1, KIP2, MTW1, PAC1, POL30, RAX2, RMI1, SGO1, SLT2, SPC98, SPH1, SRM1, STU2, SWE1, TOF1, TOP3, TUB4, YCS4* |
| GO:0045229 | External encapsulating structure organization | *ADY3, AMA1, CCW12, CDA1, CRR1, ECM12, ECM19, ECM25, ECM3, ECM33, FKS1, FKS3, FYV5, GAS1, KRE5, KRE6, NCW2, OSW1, PAU10, PAU12, PAU13, PAU15, PAU20, PAU8, PMT3, PMT5, SCW4, SLT2, SPO77, SRL1, SVP26, TIR4, TRS65, YPS6* |
| GO:0007067 | Mitotic nuclear division | *ACM1, ALK2, APC1, BUB1, CIK1, CLB4, DUO1, ECO1, GIC2, HSL7, IML3, IPL1, KDX1, KIP2, MTW1, PAC1, POL30, RMI1, SGO1, SPC98, SRM1, SWE1, TOF1, TOP3, YCS4* |
| GO:0000166 | Nucleotide binding | *ADK1, ADO1, ALK2, ARG5,6, BUB1, CCA1, CDC9, COQ8, DXO1, ERG1, FIT1, FOL1, GLK1, GRC3, GSP1, GUK1, HNT1, IME2, IPL1, KDX1, KIP2, KTI12, LCB5, MIS1, MSE1, MSW1, NRD1, ORC5, PDR12, PET54, PIF1, PMA1, PTI1, PYC2, RCK1, RFS1, RHO3, RMA1, RNP1, SLT2, SRX1, SSE2, SSK22, SVL3, SWE1, TOS3, TUB4, URA6, YEL077C, YHR218W, YLR036C, YNL247W, YRA1, YRF1-4, YRF1-5, YRF1-8* |
| GO:0022402 | Cell cycle process | *ACM1, ADY3, ALK2, AMA1, APC1, BUB1, CDA1, CDC45, CDC9, CIK1, CLB4, CRR1, DUO1, ECO1, EXO1, FKH1, FKS3, GIC2, HOP2, HSL7, IME2, IML3, IPL1, IRC19, KDX1, KIP2, MTW1, ORC5, OSW1, PAC1, POL30, PSY3, RAX2, RCK1, REC102, RED1, RFA2, RMI1, SAE3, SGO1, SLI15, SLT2, SPC98, SPH1, SPO22, SPO77, SPS4, SRM1, STU2, SWE1, TOF1, TOP3, TUB4, YCS4, YSW1* |

**Table S3. Enrichment analysis of specific down-regulated genes in the strain *ERG6*Δ.**

| ID | Term | Associated Genes Found |
| --- | --- | --- |
| GO:0043933 | Macromolecular complex subunit organization | *ABF1, AHC1, AHC2, ARP8, ASM4, ATG9, BRR2, CDC31, CEP3, COX14, COX15, DAL81, DHH1, DOC1, ESC2, ETR1, FUN19, GAL11, GIS1, IES2, LSM4, MGM1, MPS3, NPL3, NUP145, PKC1, POM34, PRP45, PRT1, PUN1, RAP1, RPS14B, RSC2, RSC4, RTG2, RVB1, SAS4, SET3, SIP1, SKP1, SNT1, SNU114, SPT6, SUP35, TFA1, TIM21, UBP13, UBX3, ULS1, VPS72, XBP1, YAP1801* |
| GO:0016071 | mRNA metabolic process | *AEP3, AI2, BI4, BRR2, CCM1, DHH1, EAP1, LSM4, NAB3, NCA2, NMD2, NPL3, PKC1, POP7, PRP45, PUF2, PUF3, RTT103, SMX3, SNU114, SPT5, SPT6, SUP35, THO2, YGR122W* |
| GO:0006996 | Organelle organization | *AEP3, ARP8, ASM4, ATG12, ATG20, ATG32, ATG9, BLI1, CCM1, CDC27, CDC31, CDC55, CEP3, COX14, DHH1, DOC1, ENV11, ENV7, ESC2, ETR1, GOS1, GYP1, IME1, IVY1, JNM1, LSM4, MDJ2, MDM34, MGM1, MHF1, MIC27, MIH1, MIP1, MMS22, MND2, MPS3, MRPL22, MRPL6, MSC3, MSS4, MTE1, NGR1, NIP100, NUP145, PAH1, PET123, PET494, PEX12, PKC1, POM34, PUF3, RAP1, RIM4, RPS14B, RSC2, RSF1, RTG2, RTS1, RVB1, SAP1, SEC20, SET3, SKP1, SNT1, SNU114, SNX41, SPT6, SRC1, SSK2, TIM21, TOM22, UGO1, VPS30, YFT2, YJR120W, YMR018W, YOR019W* |
| GO:0022402 | Cell cycle process | *ASM4, CDC27, CDC31, CDC55, CEP3, DOC1, DPB11, ESC2, FAR8, IME1, JNM1, MHF1, MIH1, MMS22, MND2, MPS3, MSA1, MSC3, MSS4, NIP100, PKC1, POM34, RIM4, RSC2, RTS1, SAP155, SET3, SKP1, SMA1, SNT1, SPO14, SPS100, SPS19, SPT6, SRC1, STE50, VHS1, XBP1* |
| GO:0005622 | Intracellular | *ABF1, ADH2, AEP3, AHC1, AHC2, AI2, AIM18, AIM41, ALT2, ALY1, APL2, APL3, ARG80, ARP8, ASM4, ATG12, ATG20, ATG32, ATG9, AVT4, BAT2, BI4, BLI1, BRR2, BSD2, CAB2, CCM1, CDC27, CDC31, CDC55, CEP3, CIT3, CMC4, CMK1, COB, COX14, COX15, COX3, CRP1, CRZ1, CSN9, DAL81, DAL82, DHH1, DOC1, DPB11, DYN3, EAP1, ECM21, EFM1, ENV11, ENV7, ERF2, ESC2, ESL2, ETR1, FAR8, FDH1, FLC3, FRA1, FUN19, FYV10, GAL10, GAL11, GAP1, GCR2, GCV1, GIS1, GLC8, GLN1, GLO4, GOS1, GPI19, GRE1, GTT1, GYP1, HAA1, HFD1, HLJ1, HVG1, HYR1, IES2, IGO2, IME1, INM2, IVY1, IXR1, JNM1, KHA1, KIN82, LEU4, LSM4, MAL13, MDJ2, MDM34, MFG1, MGM1, MHF1, MIC27, MIH1, MIP1, MMS22, MND2, MPC1, MPS3, MRPL22, MRPL6, MRS6, MSA1, MSS4, MTE1, MTR2, NAB3, NBP2, NCA2, NGR1, NIP100, NMD2, NPC2, NPL3, NRG1, NUP145, OAR1, PAH1, PCL8, PCS60, PET123, PET494, PEX12, PIG1, PIL1, PKC1, PKH3, PKP2, PLC1, POL31, POM34, POP7, POX1, PRP45, PRS1, PRT1, PSP1, PUF2, PUF3, PUN1, PXP1, RAP1, RCR1, REE1, RGR1, RIM4, RPI1, RPL39, RPL8A, RPO26, RPS14B, RSC2, RSC4, RSF1, RTC2, RTG1, RTG2, RTR1, RTS1, RTT103, RVB1, SAP1, SAP155, SAS4, SAW1, SBH2, SCH9, SEC20, SET3, SHH3, SHH4, SHM2, SIP1, SKN7, SKP1, SKP2, SMX3, SNA4, SNT1, SNU114, SNX41, SPO14, SPS100, SPS19, SPT5, SPT6, SRC1, SSK2, STB3, STE4, STE50, STE7, SUP35, TCO89, TDA9, TEC1, TES1, TFA1, TGL4, THO2, TIM21, TMA108, TOM22, TPK2, TPO5, TSC10, UBX3, UGO1, ULS1, USV1, VHS1, VNX1, VPS27, VPS30, VPS72, XBP1, YAP1801, YBR090C, YDL183C, YFT2, YHR202W, YJL133C-A, YLR345W, YMR018W, YPL109C, YPL260W, ZNF1* |
| GO:0043231 | Intracellular membrane-bounded organelle | *ABF1, AEP3, AHC1, AHC2, AI2, AIM18, AIM41, ALT2, APL2, ARG80, ARP8, ASM4, ATG32, ATG9, AVT4, BAT2, BI4, BRR2, BSD2, CAB2, CCM1, CDC27, CDC31, CDC55, CEP3, CIT3, CMC4, COB, COX14, COX15, COX3, CRP1, CRZ1, CSN9, DAL81, DAL82, DOC1, DPB11, EFM1, ENV11, ENV7, ERF2, ESC2, ESL2, ETR1, FAR8, FLC3, FUN19, FYV10, GAL11, GAP1, GCR2, GCV1, GIS1, GLN1, GLO4, GOS1, GPI19, GTT1, GYP1, HAA1, HFD1, HLJ1, HVG1, HYR1, IES2, IGO2, IME1, IVY1, IXR1, KHA1, KIN82, LEU4, LSM4, MAL13, MDJ2, MDM34, MFG1, MGM1, MHF1, MIC27, MIP1, MMS22, MND2, MPC1, MPS3, MRPL22, MRPL6, MSA1, MSS4, MTR2, NAB3, NCA2, NMD2, NPC2, NPL3, NRG1, NUP145, OAR1, PAH1, PCL8, PCS60, PET123, PET494, PEX12, PKC1, PKH3, PKP2, PLC1, POL31, POM34, POP7, POX1, PRP45, PSP1, PUF3, PXP1, RAP1, RCR1, RGR1, RIM4, RPI1, RPO26, RPS14B, RSC2, RSC4, RSF1, RTC2, RTG1, RTG2, RTR1, RTS1, RTT103, RVB1, SAP1, SAS4, SAW1, SBH2, SCH9, SEC20, SET3, SHH3, SHH4, SIP1, SKN7, SKP1, SMX3, SNA4, SNT1, SNU114, SPO14, SPS100, SPS19, SPT5, SPT6, SRC1, STB3, TCO89, TDA9, TEC1, TES1, TFA1, THO2, TIM21, TOM22, TPK2, TPO5, TSC10, UGO1, ULS1, USV1, VHS1, VNX1, VPS30, VPS72, XBP1, YBR090C, YDL183C, YFT2, YHR202W, YJL133C-A, YMR018W, YPL109C, YPL260W, ZNF1* |
| GO:0001098 | Basal transcription machinery binding | *GAL11, NPL3, RAP1, RTT103, SPT5, TFA1* |
| GO:0016627 | Oxidoreductase activity, acting on the CH-CH group of donors | *COX15, ETR1, POX1, SHH3, SHH4, SPS19, ZTA1* |
| GO:0006091 | Generation of precursor metabolites and energy | *AI2, CIT3, COB, COX3, ETR1, GCR2, GLC3, GLC8, GLG1, NCA2, OAR1, PAH1, PCL8, PIG1, PUF3, RAP1, RSF1, SHH3, SHH4, YJR120W, YLR345W* |
| GO:0006366 | Transcription from RNA polymerase II promoter | *ABF1, ARG80, CEP3, CRZ1, DAL81, DAL82, FRA1, FUN19, GAL11, GCR2, GIS1, HAA1, IME1, IXR1, MAL13, NAB3, NPL3, NRG1, PAH1, RAP1, RGR1, RPI1, RPO26, RSC2, RSC4, RSF1, RTG1, RTG2, RTR1, RVB1, SCH9, SKN7, SPS100, SPT5, SPT6, STB3, TEC1, TFA1, THO2, USV1, XBP1, YGR122W, ZNF1* |
| GO:0031323 | Regulation of cellular metabolic process | *ABF1, AHC1, ARG80, ARP8, ASM4, CDC31, CDC55, CEP3, COX14, CRZ1, DAL81, DAL82, DHH1, DOC1, DPB11, EAP1, EFM1, ESC2, FRA1, FUN19, FYV10, GAL11, GCR2, GIS1, GLC8, HAA1, IES2, IGO2, IME1, IXR1, LAG2, LRE1, MAL13, MFG1, MIH1, MPS3, MSA1, MTE1, NBP2, NCE102, NGR1, NPL3, NRG1, NUP145, PAH1, PCL8, PET494, PIG1, PIL1, PKC1, PRT1, RAP1, RGR1, RPI1, RSC2, RSC4, RSF1, RTG1, RTG2, RTR1, RTT103, RVB1, SAS4, SAW1, SCH9, SET3, SKN7, SKP1, SKP2, SNT1, SPS100, SPT5, SPT6, SSK2, STB3, STE50, STE7, TDA9, TEC1, TFA1, THO2, TMA108, ULS1, USV1, VPS72, XBP1, YGR122W, YLR345W, ZNF1* |


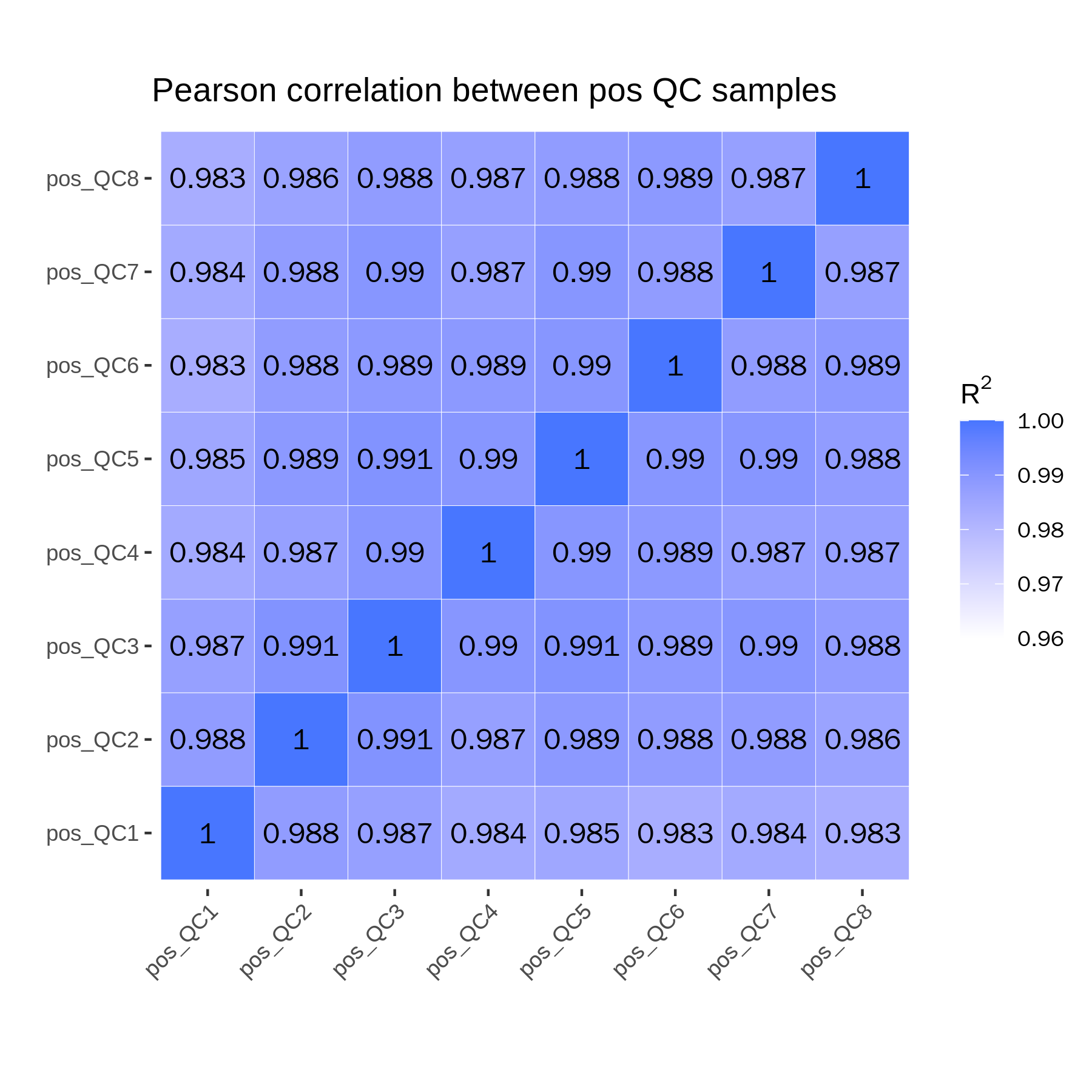


**Figure S2** **Pearson correlation analysis of positive metabolites between replicate samples**.


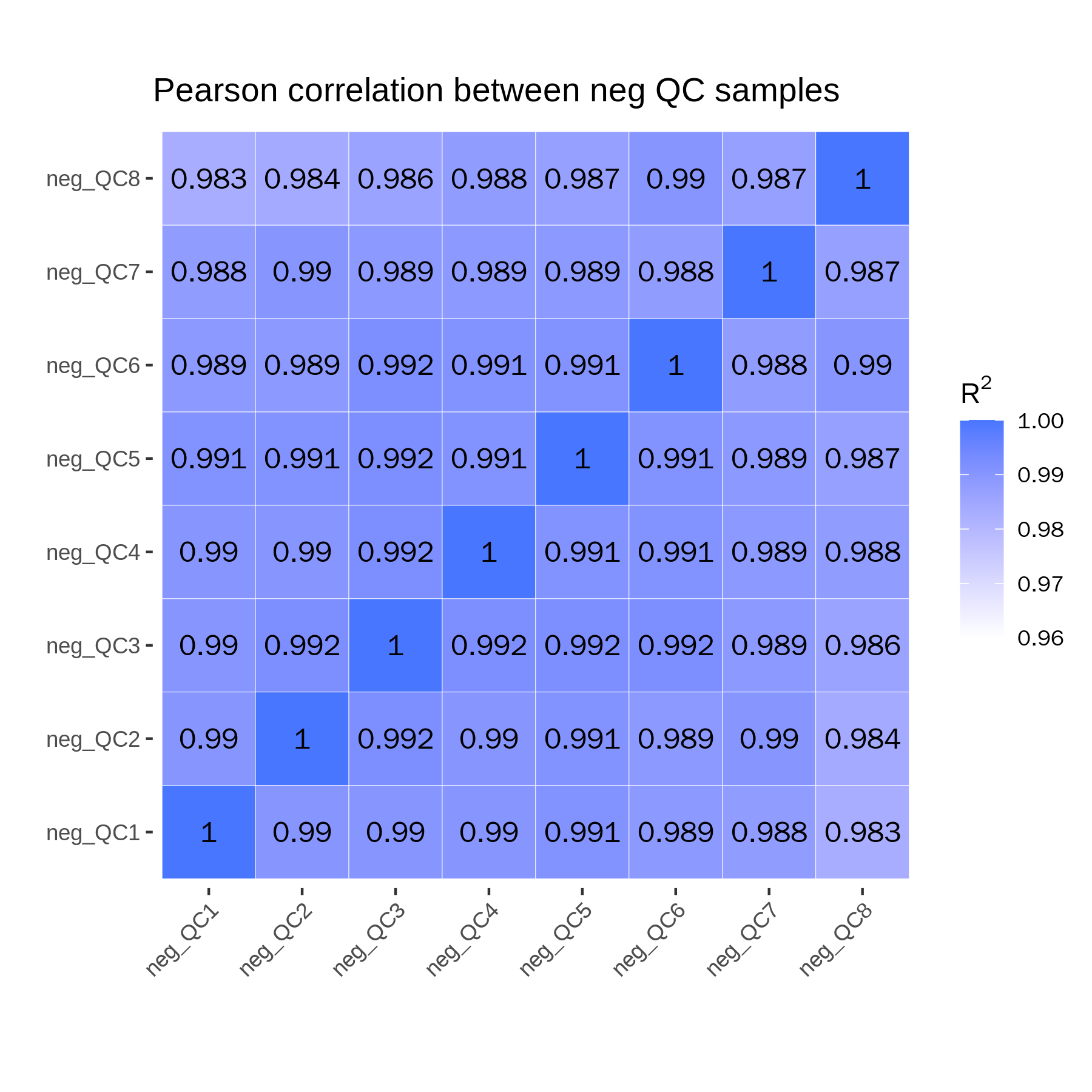


**Figure S3** **Pearson correlation analysis of negative metabolites between replicate samples**.


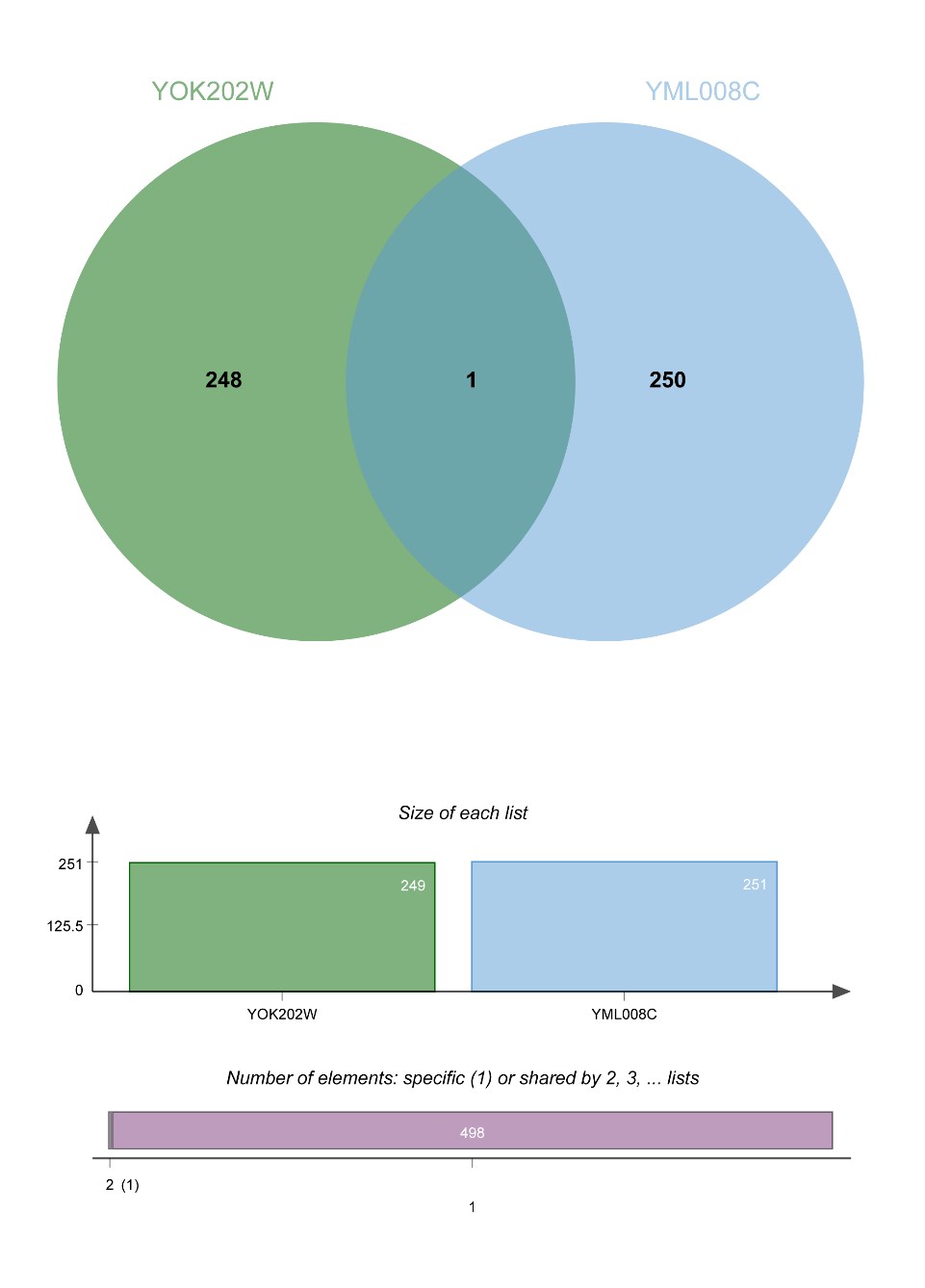


**Figure S4** **Venn diagram of the up-regulated metabolites in the strain BY4741 and *ERG6*Δ.** YOK202W represent the up-regulated metabolites in the strain BY4741 after CA treatment for 3 h. YML008C represent the up-regulated metabolites in the strain *ERG6*Δ after CA treatment for 3 h.


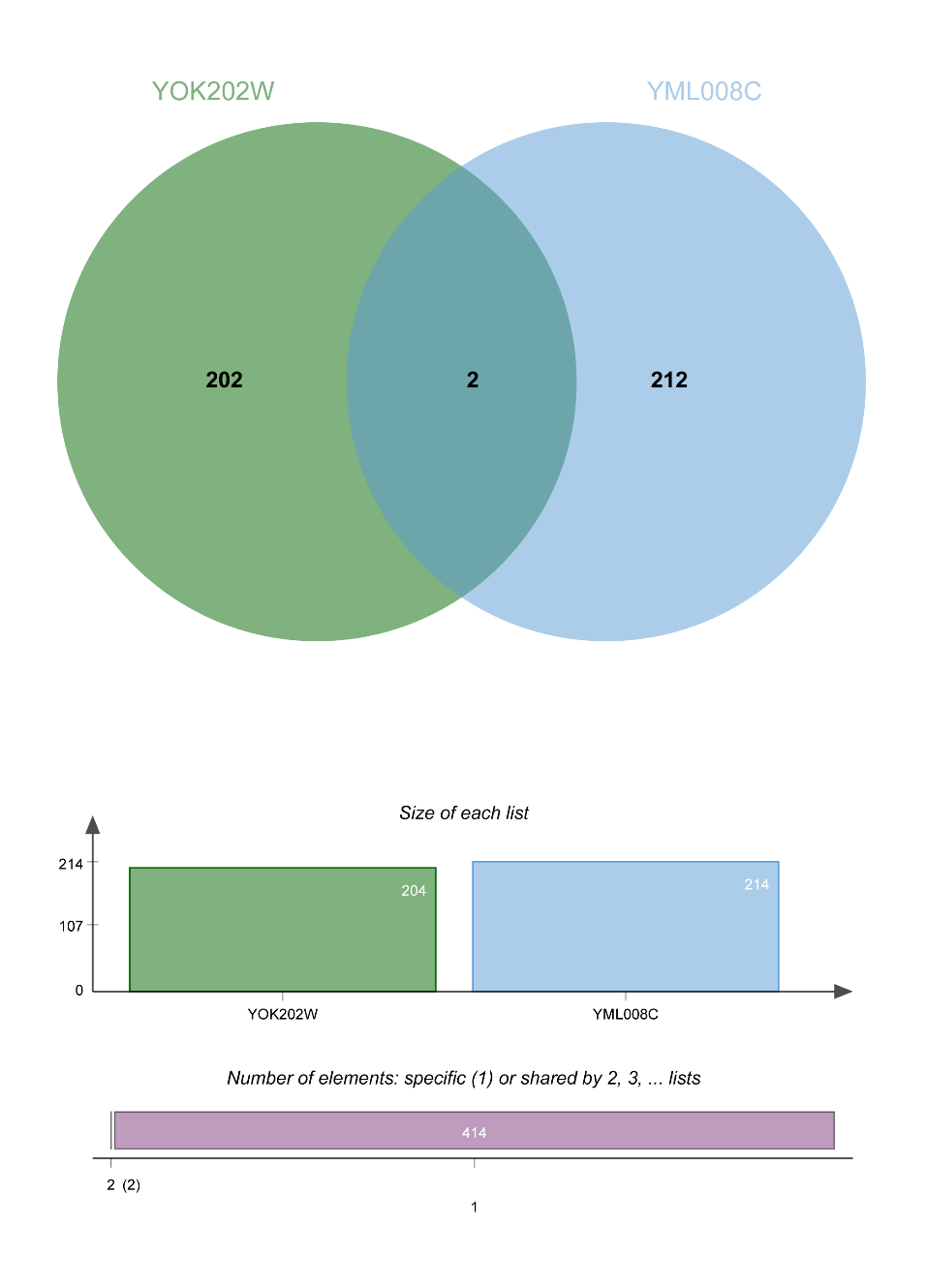


**Figure S5** **Venn diagram of the down-regulated metabolites in the strain BY4741 and *ERG6*Δ.** YOK202W represent the down-regulated metabolites in the strain BY4741 after CA treatment for 3 h. YML008C represent the down-regulated metabolites in the strain *ERG6*Δ after CA treatment for 3 h.


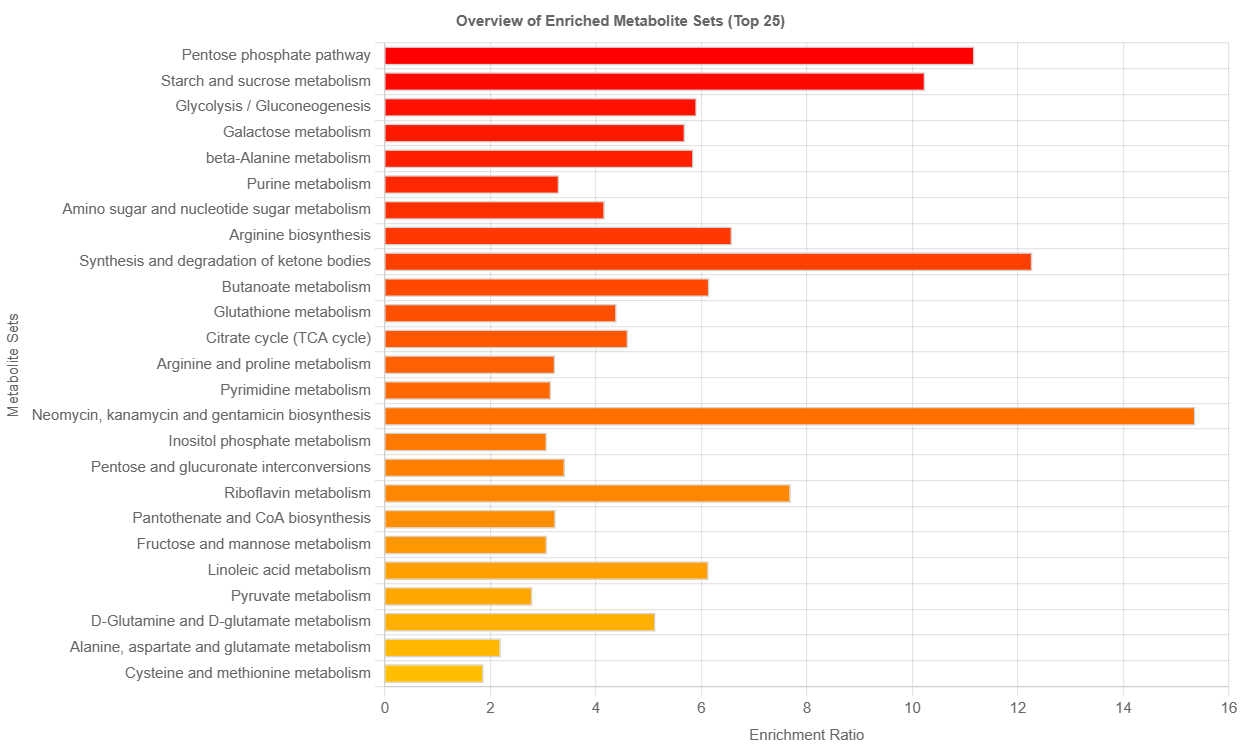

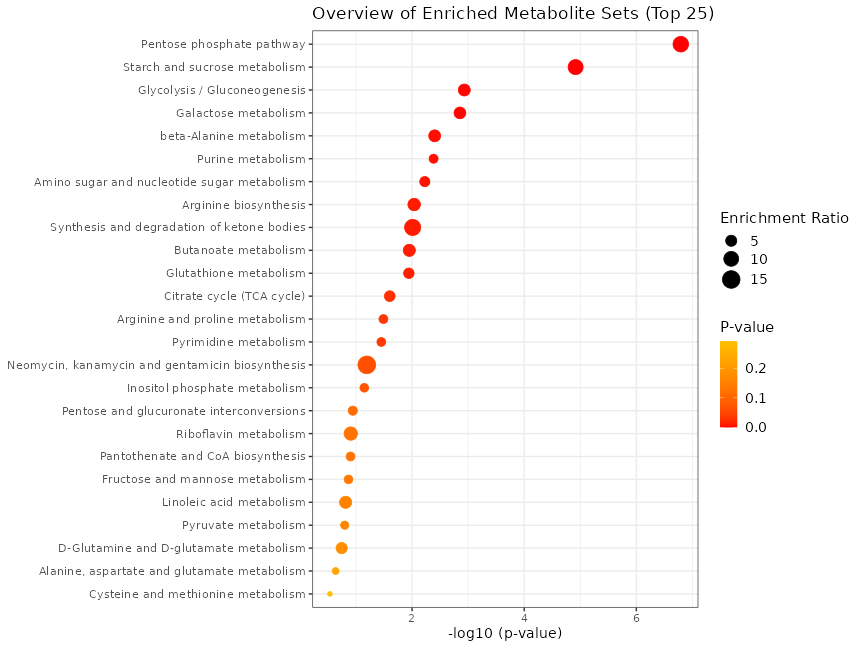


**Figure S6 Enrichment analysis of specific metabolites up-regulated in the strain *ERG6*Δ.**


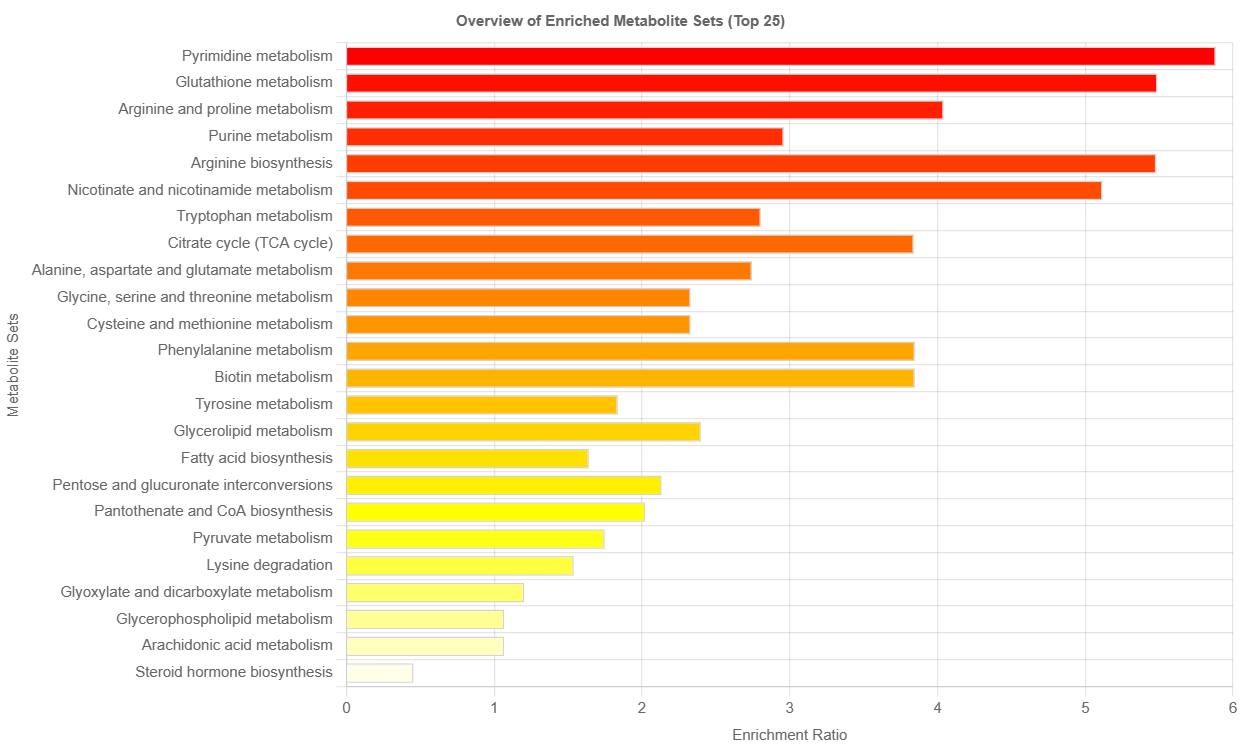

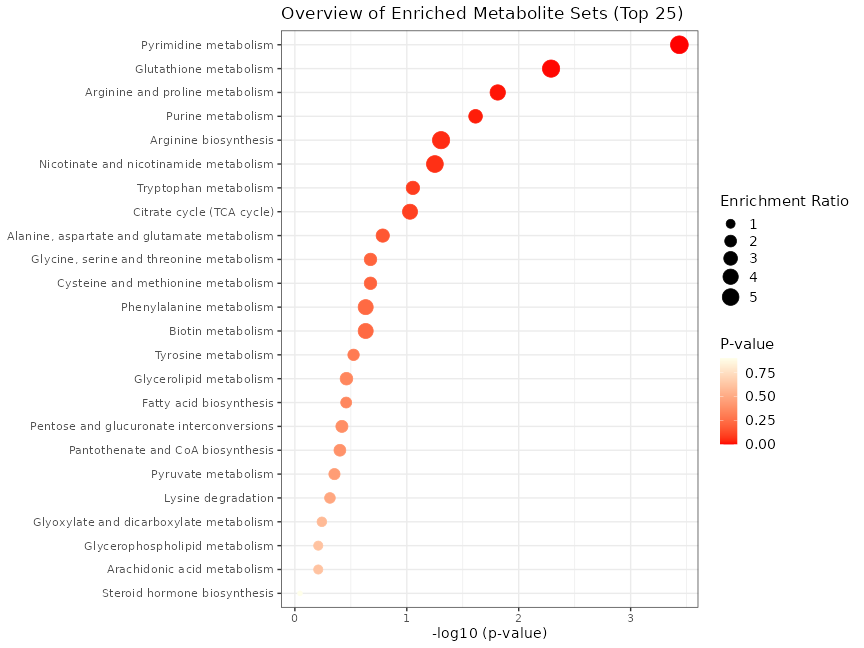


**Figure S7 Enrichment analysis of specific metabolites down-regulated in the strain *ERG6*Δ.**


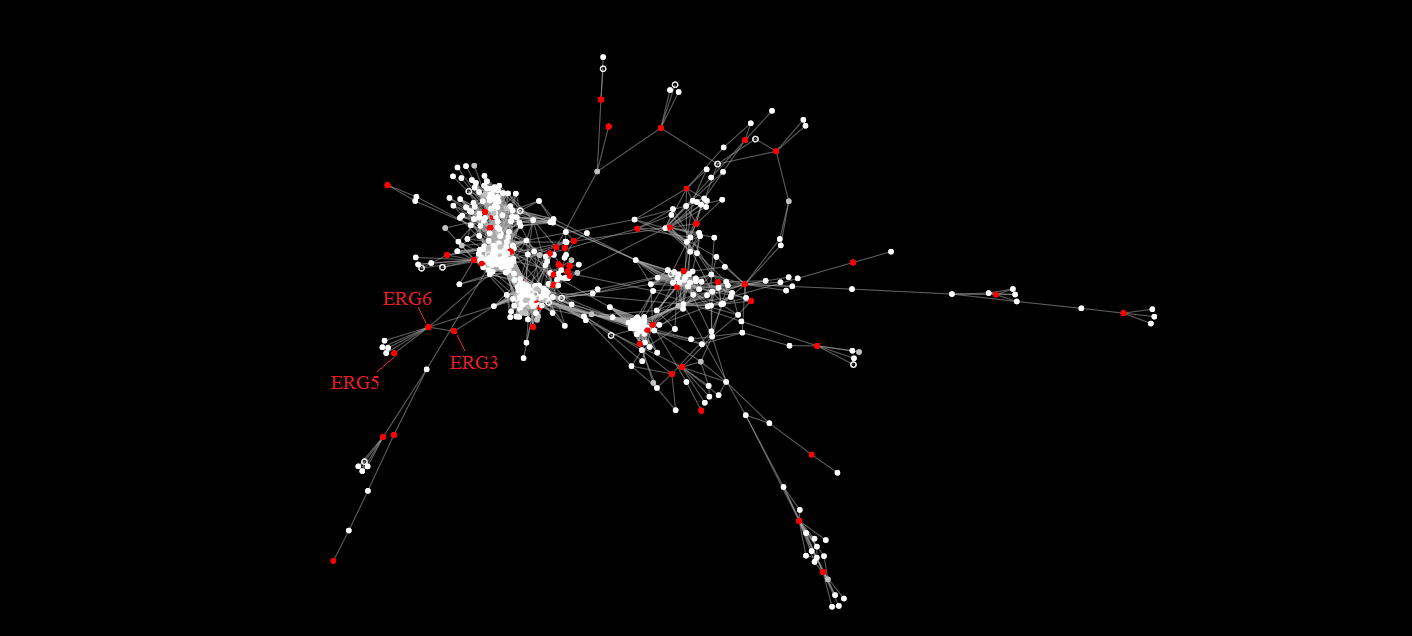


**Figure S8 An interaction analysis of up-regulated membrane genes in the strain *ERG6*Δ through TheCellMap.**


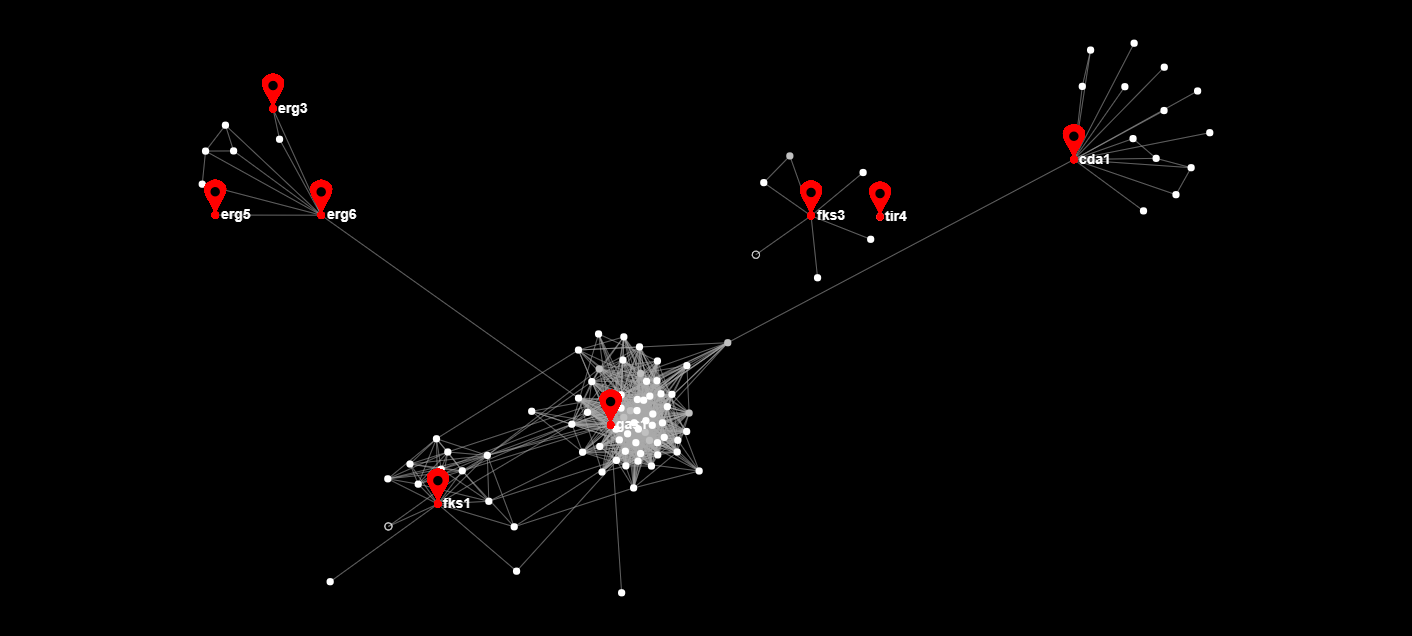


**Figure S9 An interaction analysis of down-regulated membrane genes in the strain *ERG6*Δ through TheCellMap.**
